# YBX1 modulates humoral immunity through post-transcriptional regulation in B cells

**DOI:** 10.3389/fimmu.2025.1653073

**Published:** 2025-09-10

**Authors:** Viktoria Kunz, Kathryn S. Bommert, Ralf Bargou, Kurt Bommert

**Affiliations:** Comprehensive Cancer Center Mainfranken, University Hospital Würzburg, Würzburg, Germany

**Keywords:** YBX1, lymphocyte, hematopoiesis, knockout mice, mRNA binding protein, humoral immunity

## Abstract

**Introduction:**

Y-box binding protein 1 (YBX1) is a multifunctional RNA- and DNA-binding protein implicated in transcriptional regulation, mRNA stabilization, and translation, as well as cell proliferation and stress responses. Although widely studied in cancer and inflammation, its *in vivo* role in hematopoiesis and immune regulation remains incompletely understood.

**Methods:**

To investigate YBX1 function in these contexts, we performed transplantation of fetal liver cells (FLCs) from E14.5 *Ybx1^-/-^* and *Ybx1^+/+^* embryos (CD45.2) into lethally irradiated CD45.1 congenic recipients. Hematopoietic reconstitution, immune cell subsets, B cell receptor (BCR) signaling, and humoral responses were subsequently analyzed.

**Results:**

Despite markedly reduced cellularity and progenitor frequencies in *Ybx1^-/-^* fetal livers, multilineage hematopoietic reconstitution was largely preserved. Analysis of immune cell subsets revealed functional alterations: *Ybx1^-/-^* myeloid-derived suppressor cells (MDSCs) displayed enhanced nitrite production associated with upregulated *Nos2* expression while maintaining their immunosuppressive capacity. In the lymphoid compartment, *Ybx1^-/-^* B cells underwent normal maturation and proliferated in response to LPS and IL-4, but exhibited weakened BCR signaling, characterized by reduced calcium mobilization and diminished expression of key signaling proteins, including BLNK. Strikingly, humoral responses were compromised in *Ybx1^-/-^* FLC-reconstituted mice, with significantly reduced antigen-specific antibody production following both T cell-dependent and -independent immunizations.

**Discussion:**

Mechanistic analyses demonstrated that YBX1 directly binds to immunoglobulin mRNAs and enhances their translation, establishing a post-transcriptional mechanism by which YBX1 supports humoral immunity. Together, these findings reveal an unexpected, non-redundant role for YBX1 in regulating immune effector functions. By enhancing immunoglobulin and myeloid inflammatory mediator mRNA translation, YBX1 emerges as a contributor to immune homeostasis and possibly tumor immune evasion by regulating the expression of immunomodulatory factors in immune and malignant cells alike.

## Introduction

Y-box-binding protein 1 (YBX1) is a member of the DNA- and RNA-binding protein (RBP) superfamily (1). Its cell-wide distribution reflects its involvement in diverse processes such as mRNA splicing, proliferation, mRNA packaging, and translation (2). While YBX1 has been extensively studied in the context of tumor growth, chemoresistance, and metastasis (3), its function in non-malignant hematopoietic cells remains poorly understood.

During embryogenesis, YBX1 is highly expressed in hematopoietic stem and progenitor cells (HSPCs), and *Ybx1^-/-^* mice display embryonic lethality between E14.5 and E18.5 due to multiple developmental abnormalities (4, 5). Single-cell transcriptomic and proteomic analyses consistently detect YBX1 expression in all B cell subsets, including plasma cells, in both mice and humans ((6, 7); The Human Protein Atlas (https://www.proteinatlas.org)). Its importance for B cells is supported by recent studies using a conditional *Ybx1* knockout model (*Ybx1^cko^*). These authors found reductions in peripheral B cell populations in the *Ybx1^cko^*, although the interpretation of these findings is limited by competitive repopulation and residual YBX1 expression in non-targeted cells (8). Additionally, they report no YBX1 dependence for other hematopoietic cells in the *Ybx1^cko^* model.

YBX1 has been implicated in the post-transcriptional regulation of key signaling proteins, including those involved in B cell receptor (BCR) signaling and plasma cell function (8, 9). Its capacity to bind mRNAs and enhance translation efficiency (10) suggests a possible role in regulating B cell fate decisions. This is particularly relevant because BCR-induced calcium flux is a critical driver of B cell activation, class switching, and antibody secretion (11), and disruptions in calcium signaling are known to impair immune responses in both T and B cells (12, 13).

Although YBX1 is ubiquitously expressed, its basic function in normal hematopoietic cells remains unclear. Given its involvement in numerous hematologic malignancies, elucidating its physiological role in the immune system is essential.

In this study, we addressed this question by reconstituting lethally irradiated C57BL/6 mice with fetal liver cells (FLCs) derived from either *Ybx1^-/-^* or *Ybx1^+/+^* embryos. We comprehensively analyzed lymphoid and myeloid compartments at phenotypic, functional, and molecular levels. Our findings demonstrate that YBX1 is dispensable for cell proliferation, engraftment, and basal lineage differentiation in both compartments. However, it is critical for effective antigen-specific antibody production. YBX1 loss was associated with functional alterations including increased nitrite production of myeloid derived suppressor cells (MDSC), altered BCR signaling and translational regulation of immunoglobulin transcripts, revealing a previously unrecognized roles for YBX1 in fine-tuning myeloid function and humoral immunity.

## Results

### YBX1 expression is dispensable for adult hematopoietic development

Expression analysis of YBX1 across various hematopoietic cell types revealed variable expression levels. The lowest YBX1 expression was detected in myeloid cells (Lin^-^CD11c^-^CD11b^+^) in the bone marrow and in neutrophils (Lin^-^CD11c^-^CD11b^+^Ly6G^+^) in the spleen (**Supplementary Figure S1**). Among bone marrow-derived myeloid-derived suppressor cells (MDSCs), the Ly6C^high^ population exhibited the highest YBX1 expression, whereas the Ly6C^-^ population displayed the lowest levels.

**Figure 1:**
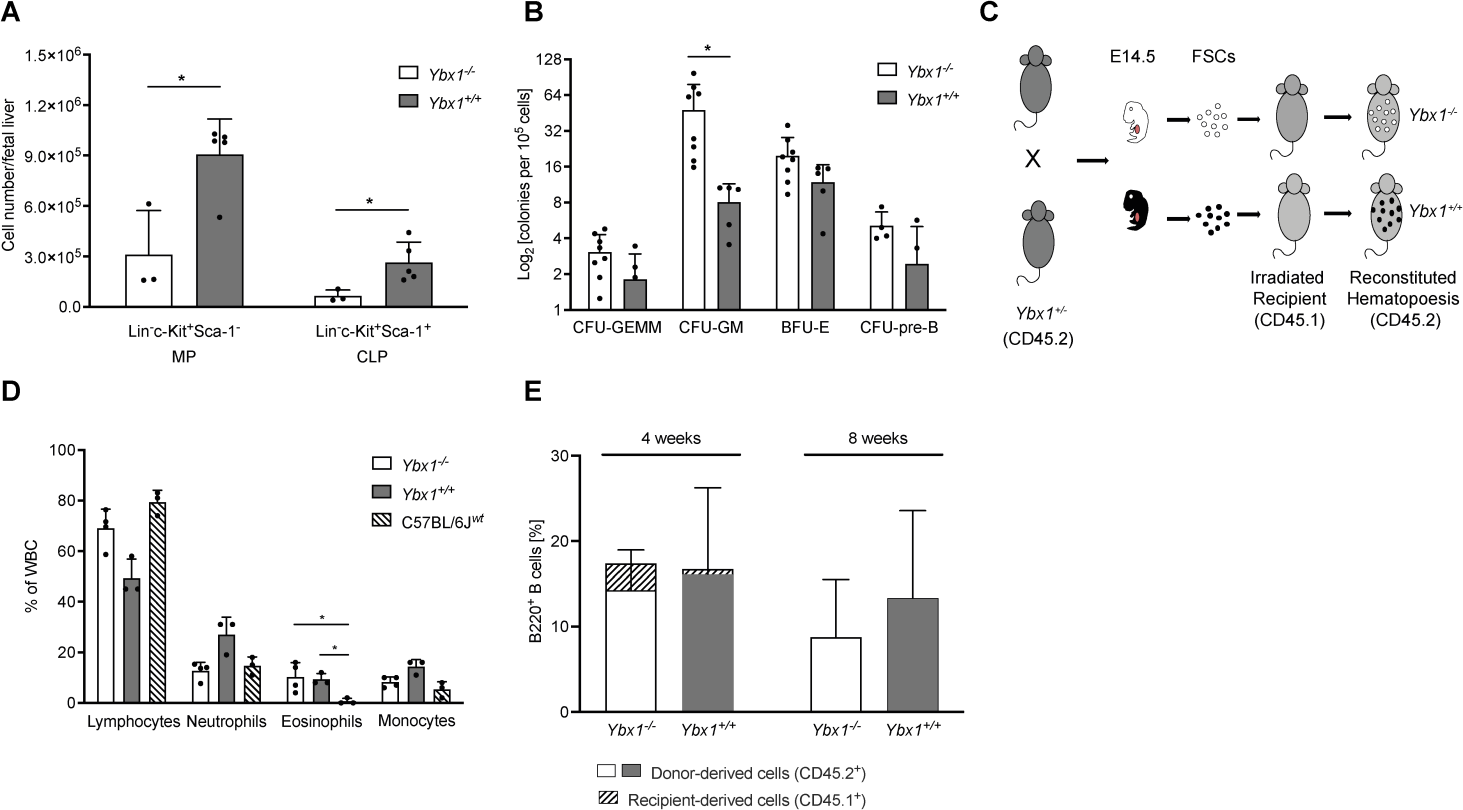
YBX1-deficient fetal liver cells fully reconstitute hematopoiesis in mice. **(A)** Progenitor Cell Numbers: The numbers of myeloid progenitors (MPs; Lin^-^, c-Kit^+^, Sca-1^-^) and common lymphoid progenitors (CLPs; Lin^-^, c-Kit^+^, Sca-1^+^) per fetal liver (E14.5) are shown as mean ± SD (n = 3–5); *p < 0.05. **(B)** Colony-Forming Assay: The colony-forming capacity of *Ybx1^+/+^* and *Ybx1^-/-^* fetal liver cells is presented as the number of colonies per 10^5^ plated cells. Colonies were classified as CFU-GEMM (granulocyte-erythrocyte-macrophage-megakaryocyte), CFU-GM (granulocyte-macrophage), CFU-pre-B (pre-B cells), and BFU-E (burst forming unit-erythroid). Columns represent mean ± SD (n = 4-8); *p < 0.05. **(C)** Schematic representation of the experimental design: Heterozygous *Ybx1^+/-^* (CD45.2) mice were bred, and at E14.5, fetal liver cells were genotyped and cultured. C57BL/6J (CD45.1) recipient mice were irradiated twice (7 Gy, four hours apart), and two hours after the second irradiation, 1.6 × 10^6^ fetal liver cells of the appropriate genotype were injected intravenously via the tail vein. **(D)** Reconstitution of the Peripheral Hematopoietic System: Frequencies of white blood cells four weeks after transplantation of *Ybx1^+/+^* or *Ybx1^-/-^* fetal liver cells. Four-week-old C57BL/6J mice served as controls. Bars indicate mean ± SD (n = 3). **(E)** Peripheral B Cell Frequencies: Frequencies of B220^+^ B cells in the peripheral blood at 4 and 8 weeks post-FLC transplantation, stratified by CD45 expression to distinguish donor-derived (CD45.2^+^) from recipient-derived (CD45.1^+^) cells.

Due to the embryonic lethality of the *Ybx1* knockout, we employed fetal liver cells (FLCs) from E14.5 *Ybx1^-/-^* and *Ybx1^+/+^* embryos (**Supplementary Figure S2**) to assess the role of YBX1 in adult hematopoiesis. *Ybx1^-/-^* fetal livers were markedly smaller and showed up to a fivefold reduction in total cellularity compared to controls (**Supplementary Figure S3**). Flow cytometric analysis revealed that *Ybx1^+/+^* fetal livers contained significantly higher numbers of common lymphoid progenitors (CLPs; Lin^-^c-Kit^+^Sca-1^+^) and myeloid progenitors (MPs; Lin^-^c-Kit^+^Sca-1^-^) than *Ybx1^-/-^* fetal livers (p < 0.05, **Figure 1A**).

To evaluate the differentiation potential of these progenitors, we conducted *ex vivo* colony-forming unit (CFU) assays. Despite the differences in overall cellularity, the frequencies of multipotent progenitors (CFU-GEMM), pre-B progenitors, and erythroid progenitors (BFU-E) were comparable between genotypes. However, granulocyte/macrophage progenitors (CFU-GM) were significantly increased in *Ybx1^-/-^* FLCs relative to *Ybx1^+/+^* (p < 0.05, **Figure 1B**).

As *in vitro* differentiation of lineage-committed progenitors appeared largely intact in the absence of YBX1, we next assessed its role in hematopoietic engraftment and reconstitution *in vivo*. Lethally irradiated C57BL/6J (CD45.1) recipient mice were transplanted with FLCs (CD45.2) from either *Ybx1^-/-^* or *Ybx1^+/+^* embryos (**Figure 1C**). Four weeks post-transplantation, except for eosinophils, the peripheral blood of both groups showed comparable distributions of lymphocytes, neutrophils, and monocytes to those in age-matched controls (p < 0.05, **Figure 1D**). At eight weeks, only donor-derived (CD45.2^+^) B220^+^ B cells were detectable in the periphery (**Figure 1E**, **Supplementary Figure S4A**), indicating a complete hematopoietic reconstitution.

Histological examination (**Supplementary Figure S4B**) confirmed normal trilineage hematopoiesis in bone marrow sections from all groups, including erythroid and myeloid progenitors as well as megakaryocytes. Splenic architecture was preserved in both genotypes, with intact white and red pulp compartments.

Collectively, these data demonstrate that despite its detectable expression in all hematopoietic subsets analyzed, YBX1 is dispensable for adult hematopoietic engraftment, differentiation, and tissue homeostasis.

### The myeloid and lymphoid compartments in detail

**Figure 2:**
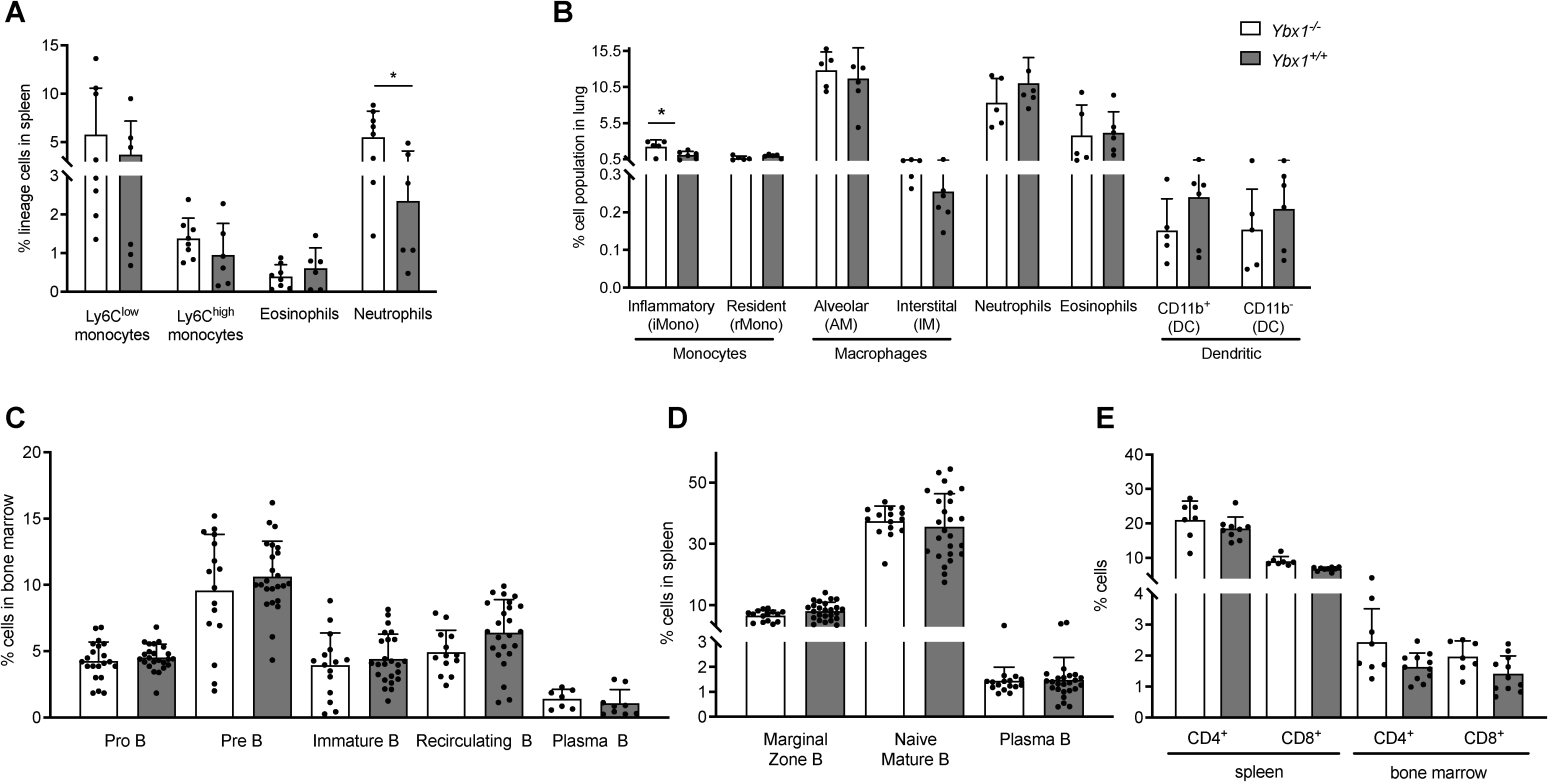
Myeloid and lymphoid compartments in reconstituted animals. **(A)** Frequencies of myeloid cell populations in the spleens of reconstituted animals (n = 6–8) *p < 0.05. **(B)** Frequencies of pulmonary myeloid populations in reconstituted animals (n = 5–6) *p < 0.05. **(C)** Frequencies of B cell subtypes in the bone marrow eight weeks after transplantation of *Ybx1^+/+^* or *Ybx1^-/-^* fetal liver cells (n = 8–24). **(D)** Frequencies of splenic B cell populations in reconstituted mice (n = 16–25). **(E)** Frequencies of CD4^+^ and CD8^+^ T cells in the spleen and bone marrow (n = 6). Each data point represents an individual mouse. Columns show mean ± SD; *p<0.05.

Histological and preliminary analyses suggested overall similarities in the major compartments of the hematopoietic system. A detailed quantitative analysis of the myeloid compartment confirmed that there were no significant differences across most cell types studied. Using established flow cytometry panels (14, 15), we analyzed myeloid populations in the spleen and lungs of FLC-reconstituted mice. In the spleen (**Supplementary Figure S5A**) eosinophil (Lin^-^CD11b^+^Ly6G^-^SSC^high^) and monocyte (Lin^-^CD11b^+^Ly6G^+^Ly6C^low/high^) numbers were unchanged (**Supplementary Table S1**). However, a significantly higher frequency of neutrophils (Lin^-^CD11b^+^Ly6G^+^, p < 0.05) was observed in *Ybx1^-/-^* FLC-reconstituted mice compared to *Ybx1^+/+^* controls (**Figure 2A**).

Lung single-cell suspensions (**Supplementary Figure S5B**) were analyzed after excluding dead cells and doublets. Using a multicolor panel to distinguish monocytes (inflammatory and resident), macrophages (alveolar and interstitial), neutrophils, eosinophils, and dendritic cell subsets (CD11b^+^ and CD11b^-^), we found that the only significant difference among pulmonary myeloid populations was an increased frequency of inflammatory monocytes in *Ybx1^-/-^* reconstituted animals (p < 0.05, **Figure 2B**).

We also performed a detailed characterization of the lymphoid compartment, beginning with stage-specific B cell subsets in bone marrow and spleen (gating strategy shown in **Supplementary Figure S6**). Briefly, pro‐B cells (B220^^+^/low^CD43^+^) and pre‐B cells (B220^lowCD43^-^^) were identified based on B220 and CD43 expression. Recirculating B cells (B220^+^IgM^+^IgD^+^) and immature B cells (B220^+^IgM^+^IgD^-^) were quantified in the bone marrow using IgM and IgD staining. In the spleen, we examined naive mature B cells (B220^+^IgM^^+^/low^IgD^+^), marginal zone B cells (B220^+^IgM^+^IgD^-^), and plasma cells (PC) (B220^^+^/^-^^CD138^+^). No significant differences were observed in the frequencies or absolute numbers of B cells in the bone marrow between *Ybx1^-/-^* and *Ybx1^^+^/^+^^* reconstituted mice (**Figure 2C**, **Supplementary Figure S7A**). Likewise, all splenic B cell subtypes were present at comparable levels across genotypes at eight weeks post-transplantation (**Figure 2D**, **Supplementary Figure S7B**, **Supplementary Table S1**). Furthermore, the B cell compartment remained stable for at least six months following reconstitution (**Supplementary Table S2**). Analysis of the T cell compartment, defined by CD4^+^ and CD8^+^ expression, also revealed no differences between *Ybx1^-/-^* and *Ybx1^+/+^* reconstituted mice (**Figure 2E**, **Supplementary Table S1**).

Collectively, these results demonstrate that reconstitution of both the myeloid and lymphoid compartments proceeds largely independent of YBX1 expression, with the only significant differences observed in pulmonary inflammatory monocytes and splenic neutrophils, both of which were elevated in *Ybx1^-/-^* FLC-reconstituted animals.

### Functional effects of YBX1 on myeloid function

**Figure 3:**
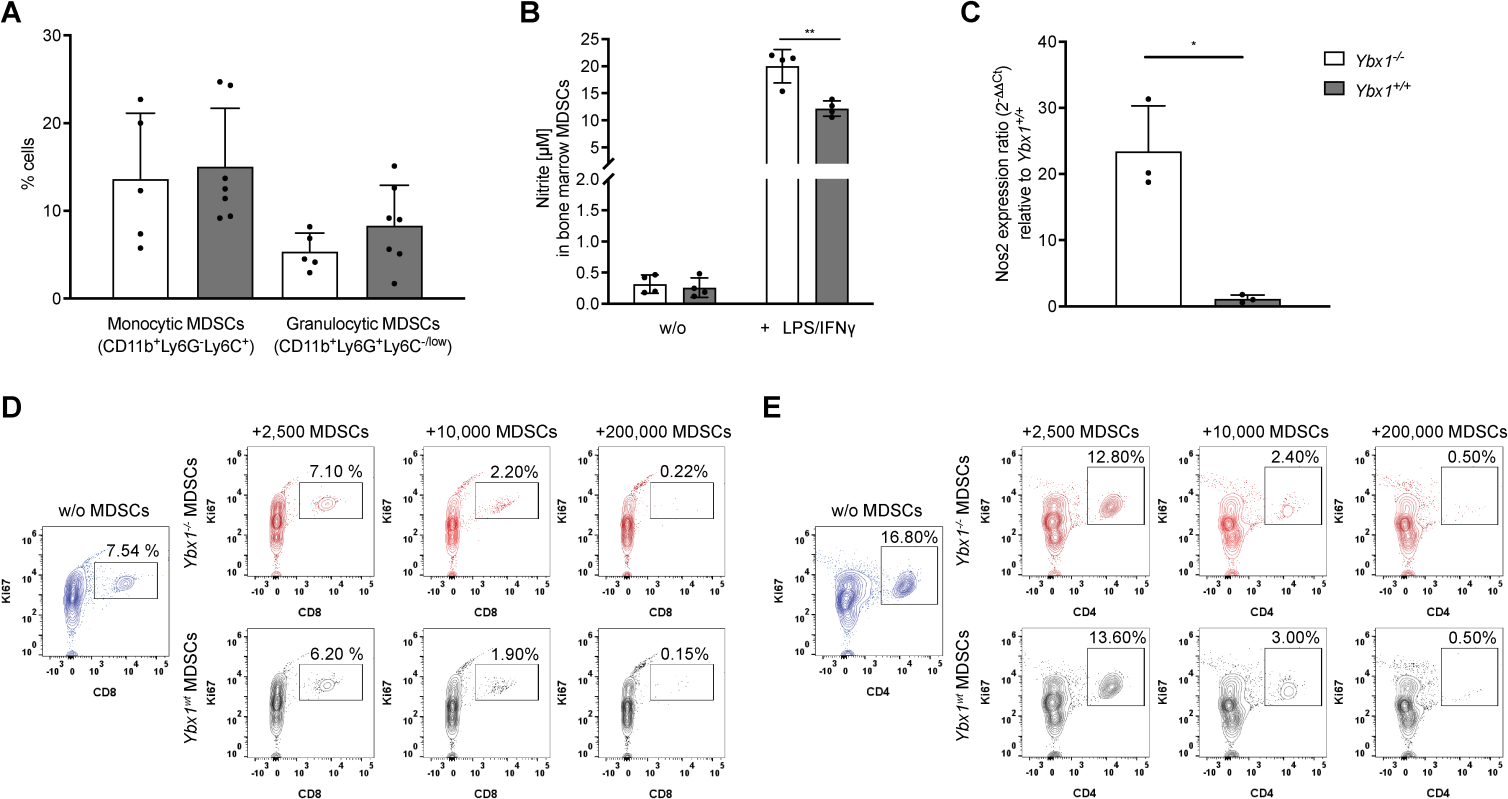
YBX1-deficient bone marrow-derived MDSCs efficiently suppress T cell proliferation *in vitro.* **(A)** Frequencies of BM-derived MDSCs after 5 days of culture with 20 ng/mL M-CSF. Data are presented as mean ± SD (n = 5–7). **(B)** Nitrite production in MDSCs after 24-hour stimulation with LPS and IFN-γ. Data are expressed as mean nitrite concentration ± SD (n = 4), **p<0.005. **(C)** mRNA levels of *Nos2* in LPS/IFN-γ stimulated *Ybx1^-/-^* and *Ybx1^+/+^* MDSCs, *p < 0.05. **(D)** CD8^+^ T Cell Suppression: Representative flow cytometry plots from two independent experiments showing Ki67^+^ CD8^+^ T cells following 4-day co-culture with 2,500, 10,000 and 200,000 MDSCs from *Ybx1^+/+^* and *Ybx1^-/-^* mice. **(E)** CD4^+^ T Cell Suppression: Representative flow cytometry plots from two independent experiments showing Ki67^+^ CD4^+^ T cells after 4-day co-culture with MDSCs.

To evaluate the functional properties of the myeloid compartment, we generated MDSCs *in vitro* as previously described (16, 17). Bone marrow cells were cultured in low-attachment plates with 20 ng/mL M-CSF to induce MDSC differentiation and expansion. After five days, cells were harvested from the supernatant and analyzed for surface marker expression. Following the exclusion of dead cells, doublets, and Lin^+^ cells, CD11b^+^ cells were further classified based on Ly6C and Ly6G expression. No differences in the frequencies of monocytic (M-MDSCs, Lin^-^CD11b^+^Ly6G^-^Ly6C^+^) and granulocytic (G-MDSCs, Lin^-^CD11b^+^Ly6G^+^Ly6C^low/^-^^) MDSCs were observed between *Ybx1^-/-^* and *Ybx1^+/+^* cultures (**Figure 3A**).

To assess effector function, MDSCs were stimulated with LPS and IFN-γ for 24 hours, followed by analysis of nitric oxide (NO) and reactive oxygen species (ROS) production. *Ybx1^-/-^* MDSCs secreted significantly higher levels of nitrite compared to *Ybx1^+/+^* controls (p < 0.005; 20 ± 3.1 µM *vs*. 12 ± 1.4 µM) (**Figure 3B**), while ROS production was independent of YBX1 (**Supplementary Figure S8**). Consistent with increased NO production, *Ybx1^-/-^* MDSCs exhibited elevated Nos2 expression (**Figure 3C**).

To test the immunosuppressive capacity of MDSCs, we co-cultured them with splenocytes under CD3/CD28 stimulation to induce T cell proliferation (18). Both *Ybx1^-/-^* and *Ybx1^^+^/^+^^* MDSCs efficiently suppressed the proliferation of wild-type CD4^+^ and CD8^+^ T cells *in vitro*, across different MDSC to T cell ratios (2,500/10,000/200,000 MDSCs; **Figures 3D, E**). Therefore, although YBX1 deficiency alters the inflammatory potential of MDSCs as reflected by increased nitric oxide production, it does not improve T cell suppression under the tested conditions.

### Reduced BCR signaling in *Ybx1^-/-^* FLC-derived B cells

Next, we evaluated the role of YBX1 in B cells by isolating splenic B cells from *Ybx1^-/-^* and *Ybx1^+/+^* FLC-reconstituted animals and stimulating them *ex vivo* for four days with LPS and IL-4. Cell proliferation was comparable between genotypes, and the frequencies of mature (B220^+^CD138^-^) and plasmablastic (B220^^+^/^-^^CD138^+^) B cells were similar (**Supplementary Figure S9A**). Following stimulation, 81–84% of cells expressed surface IgM (B220^+^sIgM^+^), and surface IgM levels were comparable between the groups (**Supplementary Figures S9B, C**), indicating that B cell maturation was unaffected by the absence of YBX1.

**Figure 4:**
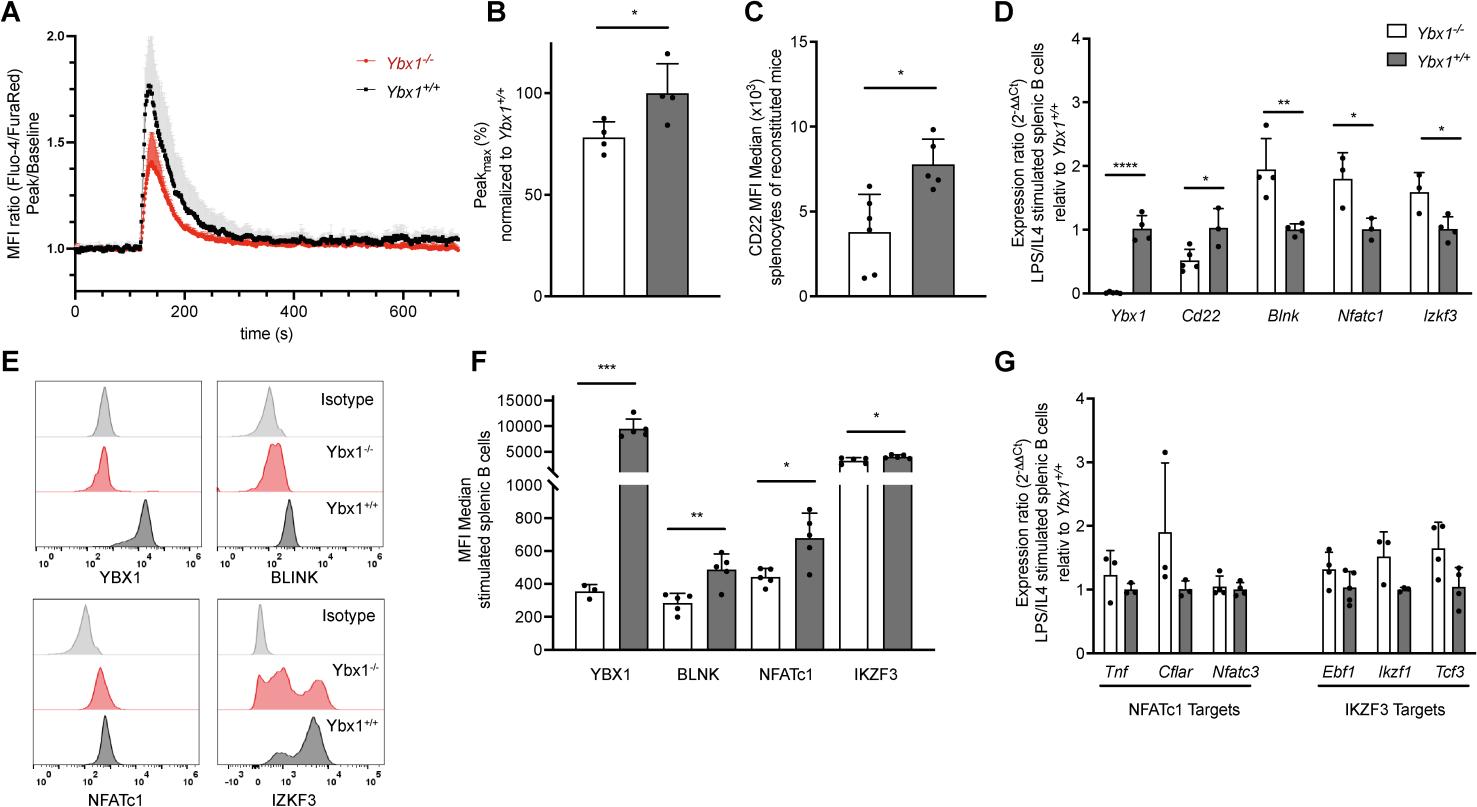
YBX1 deficiency impairs BCR signaling without affecting B cell differentiation *in vivo.* **(A)** Calcium flux in live splenic B cells stimulated with LPS/IL-4 was measured. A 2-minute baseline was recorded, followed by BCR stimulation with 25 µg/mL F(ab’)_2_ anti-IgM for 6 minutes. The MFI ratio of Fluo-4/FuraRed is shown relative to baseline for *Ybx1^+/+^* and *Ybx1^-/-^* cells over time. Each data point represents the mean ± SD from 4 independent experiments. **(B)** Maximal calcium influx following BCR stimulation, normalized to *Ybx1^+/+^* splenocytes. **(C)** Mean fluorescence intensity (MFI) for CD22 in splenocytes from *Ybx1^+/+^* and *Ybx1^-/-^* reconstituted mice. Each data point represents an individual mouse. **(D)** mRNA levels of BCR-signaling-related targets in LPS/IL-4-stimulated splenocytes, quantified using the 2^-ΔΔCt^ method. Each data point represents the relative expression ratio between *Ybx1^+/+^* and *Ybx1^-/-^* cells. **(E)** Protein Expression Profiles: Representative histograms showing the expression of YBX1, BLNK, NFATc1, and IKZF3 in splenic B cells from *Ybx1^-/-^* (red) and *Ybx1^+/+^* (black) mice; isotype controls are shown in gray. **(F)** Median fluorescence intensity for BCR-signaling targets, data presented as mean ± SD from 5 independent samples (each from an individual mouse). **(G)** Expression of Downstream Targets: mRNA expression of NFATc1 targets (*Tnf, Cflar, Nfatc3*) and IKZF3 targets (*Ebf1, Ikzf1, Tcf3*) in stimulated splenic B cells. Bars represent mean ± SD; *p<0.05; **p<0.005; ***p<0.0005; ****p<0.0001.

B cell development and function largely depend on intact BCR signaling, and therefore we looked closely at the resulting intracellular Ca^2+^ changes and the signaling proteins involved. To do so, isolated B cells were stimulated with F(ab’)_2_ anti-IgM, and downstream signaling responses were measured. Interestingly, intracellular Ca^2+^ mobilization was significantly reduced in *Ybx1^-/-^* B cells as reflected by a reduced MFI ratio of Ca^2+^-bound Fluo-4 and Ca^2+^-unbound FuraRed (**Figure 4A**). The peak Ca^2+^ amplitude was also significantly lower in *Ybx1^-/-^* cells (p < 0.05; **Figure 4B**). Notably, both CD22 mRNA and surface expression (MFI) were reduced in *Ybx1^-/-^* B cells (**Figures 4C, D**), arguing against increased CD22-mediated inhibition as the cause of diminished calcium signaling.

Additionally, mRNA levels of several critical BCR signaling genes (e.g., *Blnk*, *Nfatc1*, and *Ikzf3*, **Figure 4D**) were significantly elevated in *Ybx1^-/-^* B cells, yet their corresponding protein levels were markedly reduced (**Figures 4E, F**). To determine whether downstream signaling pathways were affected, we analyzed the expression of NFATC1 targets (*Tnf, Cflar, Nfatc3*) and IKZF3 targets (*Ebf1, Ikzf1, Tcf3*) but found no significant differences between genotypes (**Figure 4G**). These findings show that although IgM-mediated BCR signaling is reduced in *Ybx1^-/-^* B cells, compensatory mechanisms, possibly involving reduced CD22 expression, help maintain downstream transcriptional responses despite a reduction in cytoplasmic calcium mobilization upon BCR stimulation with anti-IgM.

### YBX1 is important for antigen-specific antibody production *in vitro*

**Figure 5:**
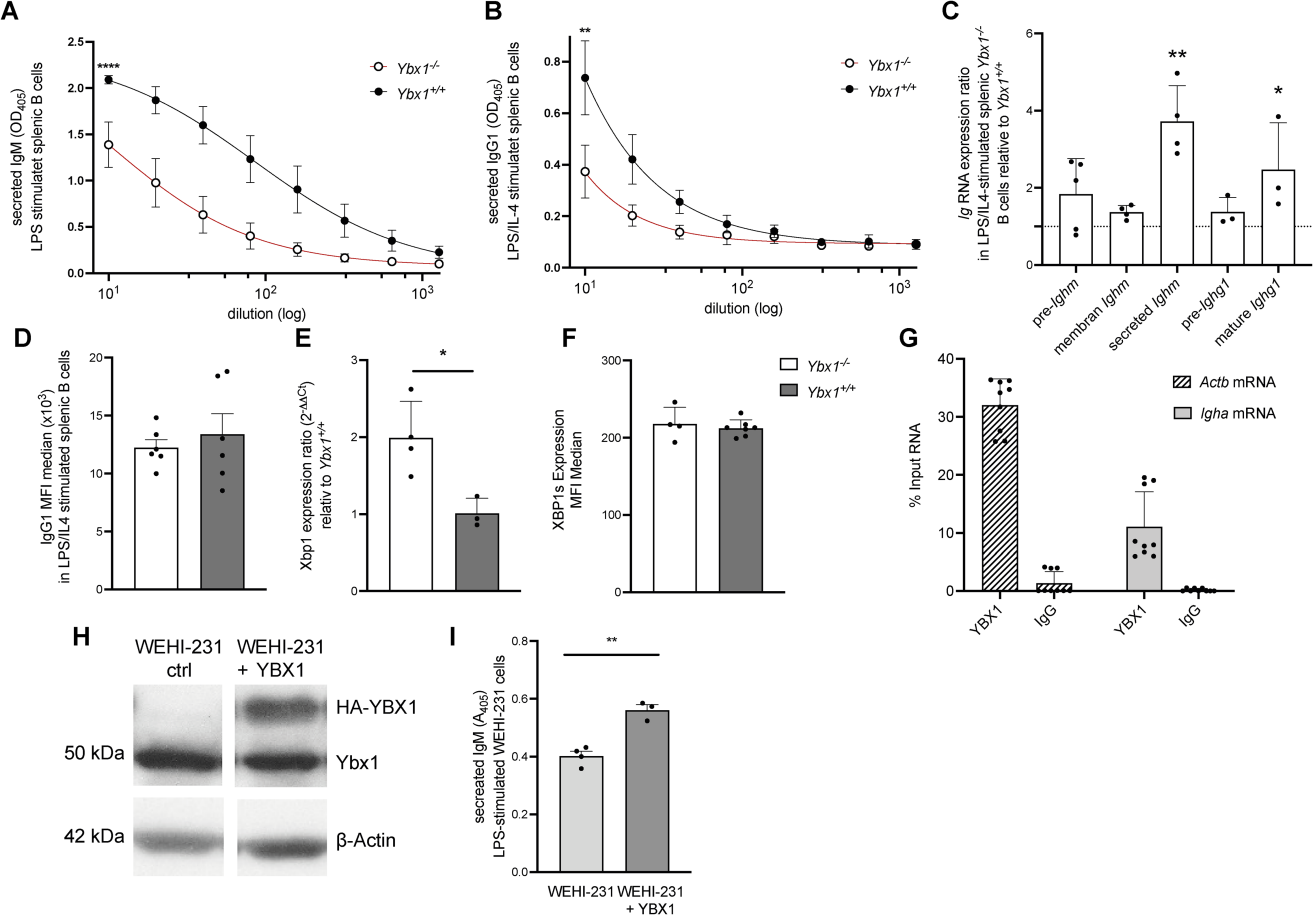
YBX1 regulates antibody production and determines the fate of mature RNA *in vitro.* **(A)** Secreted IgM: Splenic B cells from reconstituted mice were stimulated with LPS (50 μg/mL). IgM levels in culture supernatants were measured by ELISA after 4 days, ****p<0.0001. **(B)** Secreted IgG1: IgG1 levels were quantified in supernatants of B cells stimulated with LPS and mouse IL-4 (1000 U/mL) for 4 days, **p<0.005. **(C)** Immunoglobulin RNA Processing: Total RNA from stimulated splenic *Ybx1^-/-^* and *Ybx1^+/+^* B cells was analyzed for precursor and mature *Ighm* and *Ighg1* transcripts using RT-qPCR and the 2^−ΔΔCt^ method. Columns represent mean ± SD (n = 3–5); *p<0.05; **p<0.005. **(D)** Median fluorescence intensity (MFI) of intracellular IgG1 in stimulated splenic B cells. **(E)** Quantification of *Xbp1* mRNA levels in LPS/IL-4-stimulated B cells, *p<0.05. **(F)** Expression of spliced XBP1 protein (XBP1s) in B cells following stimulation, measured by intracellular staining (MFI). **(G)** YBX1 RNA Immunoprecipitation (RIP): *Actb* and *Igha* mRNA bound to YBX1 were quantified by RT-qPCR. The percentage of input RNA recovered using YBX1-specific or control IgG antibody is shown. **(H)** YBX1 Expression in WEHI-231 Cells: Western blot analysis of YBX1 expression in control and YBX1-overexpressing WEHI-231 cells; β-actin served as a loading control. **(I)** Secreted IgM in WEHI-231 Cells: IgM levels in culture supernatants of control and YBX1-overexpressing WEHI-231 cells after 4 days of stimulation with 50 μg/mL LPS, **p<0.005.

Having established stable B cell maturation despite reduced BCR signaling in *Ybx1^-/-^* B cells, we next examined the function of mature B cells, namely PC antibody secretion. Splenic B cells were stimulated with LPS for four days, and IgM secretion was measured. While total cell numbers were similar between *Ybx1^-/-^* and *Ybx1^+/+^* cultures (data not shown), IgM titers in the supernatant were significantly lower in *Ybx1^-/-^* B cells (p < 0.001; **Figure 5A**).

To assess class-switch recombination, B cells were cultured with LPS and IL-4 to induce switching to IgG1. Class-switching frequency, measured by IgG1^+^B220^+^ cells, was comparable between genotypes (**Supplementary Figure S10A**). However, *Ybx1^-/-^* B cells secreted significantly less IgG1 (p < 0.005; **Figure 5B**), indicating that while class-switching is intact, antibody secretion was significantly lower in *Ybx1^-/-^* B cells.

To explore the mechanism underlying reduced secretion, we analyzed immunoglobulin RNA processing and stability by quantifying unprocessed and mature *Ighm* and *Ighg1* transcripts and further distinguished between membrane-bound and secreted isoforms of *Ighm*. Precursor transcript levels were similar between genotypes, indicating that transcriptional initiation is unaffected by YBX1 deficiency. However, we observed a significant increase in secreted *Ighm* isoform and mature *Ighg1* transcripts in *Ybx1^-/-^* B cells (p < 0.05; **Figure 5C**). These data demonstrate that Ig RNA maturation is YBX1 independent, as similar precursor RNA levels exclude a role for YBX1 as a transcriptional regulator of Ig genes. Furthermore, the increased mature mRNA levels in *Ybx1^-/-^* B cells argue against the involvement of YBX1 in RNA stabilization.

To investigate whether defective secretion might explain reduced antibody titers, we assessed if YBX1 impacts secretion by using stable Gaussia luciferase (GLuc)-expressing mouse embryonic fibroblasts (MEFs) derived from *Ybx1^+/+^* and *Ybx1^-/-^* embryos. Comparable GLuc activity in the supernatants of *Ybx1^+/+^* and Ybx1-/- MEFs after 72 hours of incubation (**Supplementary Figure S10B**) excluded impaired secretion as a contributing factor to the reduced antibody titers. Moreover, intracellular flow cytometry analysis for IgG1 in stimulated splenic B cells revealed no intracellular accumulation (**Figure 5D**), supporting the conclusion that the defect in Ybx1-/- B cells lies at the level of translation of secretory Ig transcripts rather than transcription, splicing, or secretion.

XBP1 is essential for PC antibody production (19), and thus changes in protein levels could be responsible for the decreases in antibody production. However, in our cells *Xbp1* mRNA levels were significantly increased in stimulated *Ybx1^-/-^* B cells (p < 0.05; **Figure 5E**), but XBP1s protein levels were unchanged (**Figure 5F**), suggesting that Ybx1 may influence *Xbp1* transcription but not its splicing or translation. To further assess this, we analyzed XBP1s protein expression following ER stress induction with 2 µg/ml tunicamycin in a CRISPR/Cas9-generated Ybx1-deficient MOPC315.BM cell line. Consistent with our findings in primary B cells, XBP1s levels were comparable between *Ybx1^-/-^* and *Ybx1^+/+^* cells (**Supplementary Figure S11**). Thus, the reduced antibody production observed in *Ybx1^-/-^* B cells results neither from altered XBP1 expression nor the availability of subsequent spliced isoforms.

To determine whether YBX1 directly binds immunoglobulin RNA, we performed RNA immunoprecipitation (RIP) in two murine B cell lines: MOPC315.BM, which expresses YBX1 and secretes DNP-specific IgA, and in WEHI-231, a mature B cell line that secretes IgM. RIP followed by RT-PCR revealed that in MOPC315.BM ~30% of input *Actb* mRNA (a known YBX1 target) and ~11% of *Igha* mRNA were precipitated using an anti-YBX1 antibody (**Figure 5G**). In WEHI-231 cells, ~4% of *Actb* and ~2% of *Ighm* mRNA were recovered in the YBX1 pull-down fraction (**Supplementary Figure S12**). These results indicate that YBX1 binds to both *Igha* and *Ighm* mRNAs, albeit less efficiently than *Actb*, and suggest a direct role for YBX1 in regulating Ig RNA fate at the translational level.

To confirm this role, we overexpressed YBX1 in the WEHI-231 B cell line and stimulated the cells with LPS. YBX1 overexpression had no effect on cell proliferation (**Figure 5H**, **Supplementary Figure S13**) but significantly increased IgM secretion (p < 0.005; **Figure 5I**, **Supplementary Figure S14**), reinforcing its enhancing role in antibody production.

Collectively, these findings demonstrate that YBX1 is dispensable for B cell maturation and class switching but is important for efficient antigen-specific antibody production. The underlying mechanism likely involves immunoglobulin mRNA translation efficiency, mediated by direct interaction of YBX1 with Ig transcripts.

### Decreased T cell-dependent and T cell-independent immune responses in *Ybx1^-/-^* reconstituted mice

**Figure 6:**
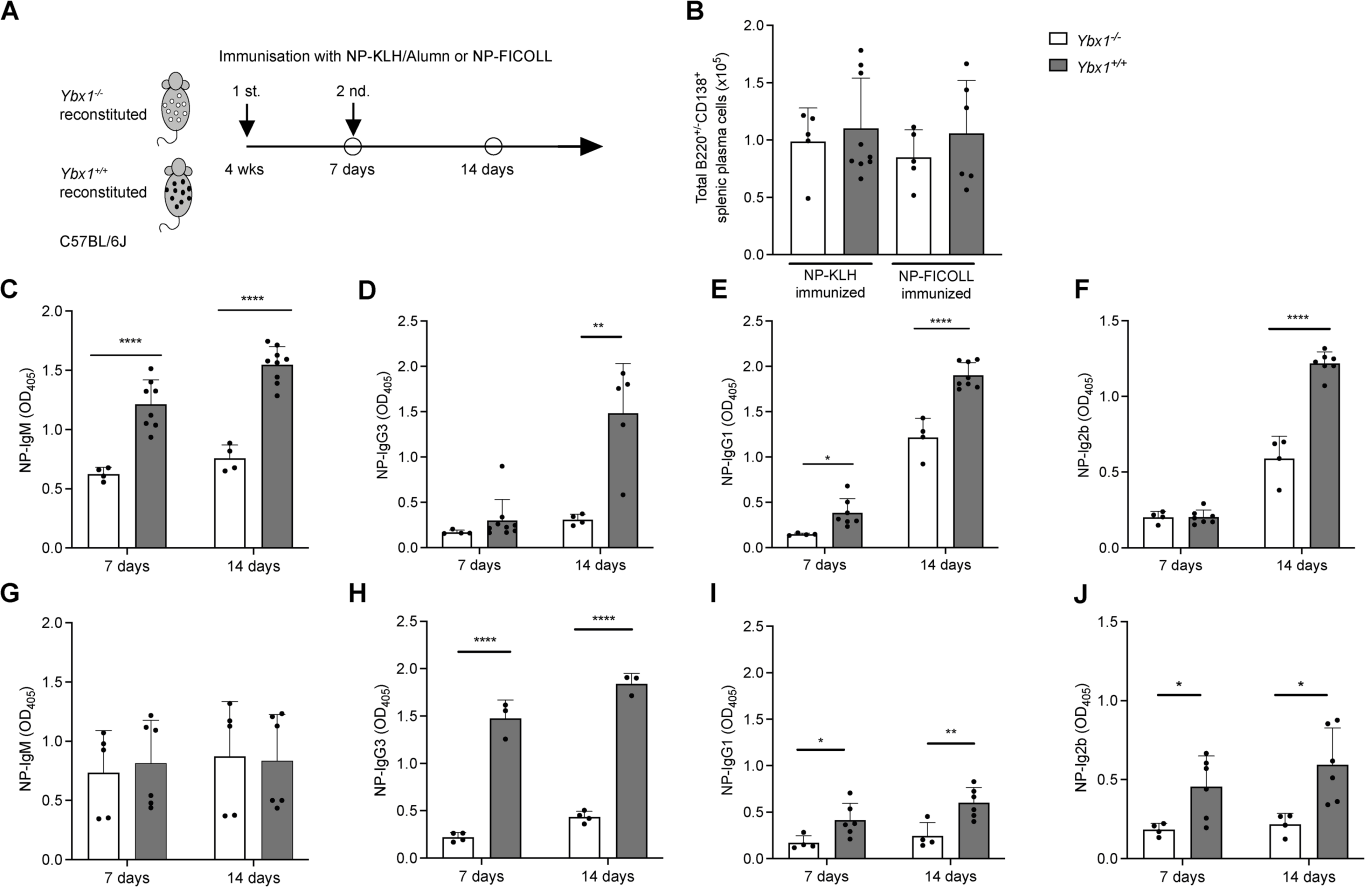
Attenuated humoral immune responses in *Ybx1^-/-^* reconstituted mice. **(A)** Experimental Design: *Ybx1^-/-^* and Ybx1^+/+^ FLC-reconstituted mice were immunized twice at one-week intervals with either NP-KLH (adjuvanted with alum) or NP-FICOLL. Sera were collected on days 7 and 14. The experiments concluded 7 weeks after FLC reconstitution. **(B)** Spleen Cellularity: Total cell numbers in the spleens of immunized animals were measured 7 days post-immunization. Bars represent mean ± SD (n = 3–9 mice). **(C–F)** Ig titers in sera from NP-KLH–immunized mice (with alum) on day 7 and day 14. **(G–J)** Ig titers in sera from NP-FICOLL–immunized mice on day 7 and day 14. Bars represent mean values ± SD, with each data point representing an individual mouse (n = 3–9). Statistical significance is indicated as follows: *p < 0.05, **p < 0.005, ***p < 0.0005, ****p < 0.0001.

To evaluate the role of YBX1 in humoral immunity *in vivo*, we immunized *Ybx1^-/-^* and *Ybx1^+/+^* FLC-reconstituted mice (four weeks post-transplantation) with either nitrophenyl (NP)-KLH, a T cell-dependent (TD) antigen, or NP-FICOLL, a T cell-independent (TI) antigen. Sera were collected one week after the initial immunization (day 7) and one week after the second antigen exposure (day 14, **Figure 6A**).

*Ybx1^-/-^* mice exhibited significantly reduced NP-specific TD Ig responses across multiple isotypes following primary immunization, including IgM (p < 0.0001), IgG1 (p < 0.05), and IgG2b (p < 0.05), while IgG3 levels remained unaffected (**Figures 6C-F**). After the second immunization, NP-specific Ig titers were further diminished in *Ybx1^-/-^* mice, including IgM (p < 0.0001), IgG3 (p < 0.005), IgG1 (p < 0.0001), and IgG2b (p < 0.0001), compared to *Ybx1^+/+^* controls. Importantly, total numbers of splenic plasma cells (B220^+^/CD138^+^) and the distribution of B cell subsets were comparable between groups (**Figure 6B**, **Supplementary Table S3**), indicating that impaired antibody responses were not due to differences in B cell numbers.

To assess T cell-independent responses, we immunized animals with NP-FICOLL and measured NP-specific antibody titers. Seven days after immunization, NP-specific IgM titers were comparable between *Ybx1^-/-^* and *Ybx1^+/+^* mice, whereas IgG3 (p < 0.0001), IgG1 (p < 0.05), and IgG2b (p < 0.05) titers were significantly lower in Ybx1^-/-^ animals (**Figures 6G-J**). Following second antigen exposure, NP-specific IgM titers remained unchanged across genotypes (**Figure 6G**, **Supplementary Figure S15**), but reductions in IgG1 (p < 0.001), IgG3 (p < 0.005), and IgG2b (p < 0.05) were again observed in *Ybx1^-/-^* mice (**Figures 6H-J**).

Together, these findings demonstrate that YBX1 is essential for robust antigen-specific antibody production during both T cell-dependent and T cell-independent immune responses *in vivo*.

## Discussion

This study provides a comprehensive analysis of YBX1 function in hematopoiesis, immune cell biology, and humoral immunity. We show that although YBX1 is broadly expressed across myeloid and lymphoid lineages, it is not essential for engraftment or quantitative hematopoietic reconstitution. However, we identify a novel and important role for YBX1 in the regulation of antigen-specific antibody production.

Our finding that YBX1 is dispensable for hematopoiesis was unexpected. Despite normal timing of hematopoietic onset, *Ybx1^-/-^* fetal livers and several other organs exhibited approximately 50% fewer cells compared to wild-type counterparts (4, 5). YBX1 has been implicated in supporting proliferation across malignant and non-malignant cell types (2), yet we observed no proliferation defects in *Ybx1^-/-^* versus *Ybx1^+/+^* hematopoietic cells *in vivo* or *in vitro*. Similarly, genetic deletion of *Ybx1* in the MOPC315.BM myeloma cell line did not alter proliferation rates (**Supplementary Figure S16**). These findings suggest that YBX1’s proliferative role is restricted to early embryogenesis (E0–E13), a critical developmental window after which most *Ybx1^-/-^* embryos fail to survive. Beyond this stage, YBX1 appears dispensable for cellular proliferation in the hematopoietic system.

Previous studies suggest that YBX3 may compensate for YBX1 deficiency, as both bind similar sets of mRNAs in HEK293 cells (20). Indeed, dual knockout of *Ybx1* and *Ybx3* delayed hematopoietic onset at E13.5, implying partial redundancy. However, YBX3 expression diminishes post-E17.5 and becomes restricted to the testis (4). Transcriptional profiling demonstrated YBX3 expression in a subset of pre-BCR cells and suggested that in this population it may support proliferation during the pre-BCR cell expansion (7). However, they also show that the contribution of YBX3 is minor relative to the reciprocal regulation between EBF1 and MYC.

The adult hematopoietic niche may also provide extrinsic compensatory cues. IL-7 signaling, via PI3K and STAT pathways, is known to support B cell survival and proliferation (21, 22). Indeed, IL-7–stimulated cultures from both *Ybx1^-/-^* and *Ybx1^+/+^* FLCs yielded similar frequencies of B220^+^IgM^+^ cells (**Supplementary Figure S17**), supporting the idea that IL-7 can offset YBX1 loss. Moreover, the increased granulocyte/macrophage progenitors (CFU-GM) in *Ybx1^-/-^* FLCs may reflect a compensatory mechanism to sustain hematopoiesis. Elevated neutrophils and inflammatory monocytes in *Ybx1^-/-^* reconstituted mice could enhance B cell activity via secretion of BAFF or APRIL (23, 24), further stabilizing immune function despite YBX1 deficiency.

YBX1 deletion also affected myeloid-derived suppressor cells (MDSCs), which showed increased nitrite production likely due to Nos2 upregulation yet displayed no enhancement in immunosuppressive capacity. Given that nitric oxide suppresses T cell activation, this outcome was surprising. One explanation may be that IL-7–mediated compensatory signaling also modulates myeloid function. Alternatively, the suppression assay employed may not have captured the full impact of NO elevation. Co-culture models involving T cells, MDSCs, and malignant cells could provide a more nuanced understanding of these immunoregulatory dynamics.

In contrast to its limited impact on hematopoietic cell development, YBX1 emerged as a novel regulator of IgM-mediated BCR activation. Although *Ybx1^-/-^* B cells exhibit normal maturation and proliferation in response to LPS and IL-4 stimulation, they show significantly reduced ER calcium release upon BCR stimulation (**Figure 4**). This impaired calcium flux is consistent with the reduced protein levels of key signaling molecules such as Blnk, Nfatc1, and Ikzf3 observed in Ybx1-/- B cells. However, phosphorylated PLCγ2 protein levels as determined by mean fluorescence intensity after BCR stimulation were comparable between genotypes (**Supplementary Figure S18**). The decreased Ca^2+^ signaling had no impact on B cell development despite its documented importance (25–27). This is consistent with previous findings; for example, genetic ablation of both *Stim1* and *Stim2* genes, which represent critical sources of extracellular Ca^2+^ influx, did not affect B cell development or Plasma cell Ig secretion *in vivo* (13). Although these authors reported decreased IL-10 production due to the loss of NFAT signaling, our data showed that despite decreases in NFATC1, there were no changes in the expression of the target genes we studied.

We suggest compensatory cellular mechanisms made systemwide gain adjustments, thus making this decreased Ca^2+^-amplitude as effective as that of wild-type systems in BCR function and B cell development. One possible example may be CD22, a negative regulator of BCR signaling (28), which is expressed at lower levels in *Ybx1^-/-^* B cells. This decreased CD22 expression may partially offset the reduced calcium flux, thus leaving B cell proliferation and maturation intact. The notion of compensatory mechanisms circumventing the deleterious effects of decreased BCR-dependent Ca^2+^ response has also been described. For example, the co-stimulation of CD40 and TLR9 was sufficient to promote B cell survival and maturation despite weak BCR-dependent Ca^2+^ amplitude (29).

The *in vitro* and *in vivo* experiments presented here demonstrate that YBX1 is essential for efficient antibody production. *In vitro*, secreted immunoglobulin levels were significantly reduced in *Ybx1* ^-/-^ splenic B cells stimulated with LPS and IL-4, despite normal class-switch recombination (**Figure 5**). One potential explanation could involve XBP1, a transcription factor whose splicing into the active form (XBP1s) is critical for immunoglobulin production and the establishment of the secretory plasma cell phenotype (19, 30). Although *Xbp1* mRNA levels were significantly elevated in *Ybx1* ^-/-^ B cells, levels of the spliced XBP1 protein (XBP1s) remained unchanged, thereby excluding a role for XBP1s in the observed antibody production defect.

Our data instead point to a post-transcriptional role for YBX1 in regulating immunoglobulin mRNA translation. First, precursor Ig transcripts levels were comparable between genotypes, indicating unaffected transcription. Second, membrane-bound IgM mRNA and surface expression levels were also unaltered. However, secreted *Ighm* and mature *Ighg1* transcripts were significantly elevated in *Ybx1^-/-^* cells, consistent with impaired translation or secretion. Supporting this, we detected co-immunoprecipitation of YBX1 with both *Igha* and *Ighm* mRNA. Furthermore, YBX1 overexpression in WEHI-231 cells led to increased IgM secretion without altering *Ighm* transcript levels (**Supplementary Figure S19**). Together, these results highlight a role for YBX1 in specifically promoting the translation of secreted immunoglobulin mRNAs, an exciting finding that aligns with its established role in mRNA translational regulation (31).

The data presented here is consistent with broader findings that translational regulation, rather than transcription, determines protein abundance (32). It also aligns with our previous report implicating YBX1 in MYC mRNA translation in murine and human myeloma models (33), a role later supported by others (8, 34, 35).

Although YBX1 has also been implicated in mRNA splicing, particularly in cancer models where phosphorylated YBX1 alters ERK signaling via splicing regulation (36), our results exclude a splicing role in this context. We observed no differences in precursor immunoglobulin mRNA isoforms between genotypes, while mature immunoglobulin mRNA levels were even elevated in *Ybx1^-/-^* splenocytes. Moreover, secretory pathway integrity remained intact: *Ybx1^-/-^* B cells showed no intracellular Ig accumulation, and luciferase activity in the medium of Ybx1-deficient MEFs transduced with secreted Gaussia luciferase was comparable to that observed in Ybx1-proficient cells.

Overall, our findings underscore that YBX1 functions as a multifunctional regulator within the mature immune system. Although YBX1 is not required for the quantitative reconstitution of hematopoietic compartments, its loss affects qualitative aspects of hematopoietic function and is particularly critical for the complete competence of plasma cells. The impaired BCR signaling, coupled with defective post-transcriptional regulation of key signaling proteins, was largely compensated for in the absence of YBX1. However, this compensation did not extend to humoral antibody responses. Here, we clearly demonstrate a role for YBX1 in regulating mRNA translation for optimal antibody production within the adult hematopoietic system. These results have significant implications for our understanding of immune regulation and suggest a potential role for YBX1 in supporting tumor cell survival through enhanced translation of immunomodulatory factors.

## Methods

### Animals

From C57BL/6J-Ybx1 mice, a kind gift from T. Ley (Washington University, St. Louis, USA), we retrieved embryos of Ybx1^+/+^ and Ybx1^-/-^ genotypes. Additionally, 8- to 10-week-old female C57BL/6J (CD45.1) mice (Charles River, Germany) were used as recipients for transplantation experiments. All mice were bred and housed in the Center for Experimental Molecular Medicine, and experiments were done in compliance with German animal welfare regulations.

### Harvesting and transplantation of fetal liver cells

Pregnant mice were euthanized, and E14.5 fetal livers were harvested under sterile conditions. Livers were mechanically dissociated using a 40-µm cell strainer (Greiner Bio-One, Germany) and suspended in IMDM medium (Thermo Fisher, Germany) supplemented with 20% (v/v) BIT 9500 Serum Substitute (Stemcell Technologies, Canada), 100 U/mL penicillin-streptomycin, and 0.1 µM β-mercaptoethanol (Merck, Germany).

Recipient C57BL/6J (CD45.1) mice underwent lethal total-body irradiation (TBI) using a Faxitron CP-160 X-ray irradiator (Faxitron X-Ray, USA). Irradiation was administered in two fractions of 7 Gy at a 4 hour interval. Two hours following the second irradiation dose, 1.6x10^6^ fetal liver cells were injected intravenously via the tail vein in a total volume of 150 µL DPBS (Thermo Fisher, Germany). To prevent infections, transplanted mice received neomycin sulfate (2 g/L, Betapharm, Germany) in their drinking water.

### Immunization

Four weeks post-transplantation, mice were immunized intraperitoneally with 50 µg NP-KLH (LGC, Biosearch/Biocat Germany) in 500 µL Imject Alum Adjuvant (Thermo Fisher Germany, 1:1 diluted in DPBS) to induce a T cell-dependent (TD) immune response or 50 µg NP-FICOLL (LGC, Biosearch/Biocat Germany) in 100 µL DPBS to assess T cell-independent (TI) immune responses.

### Functional, biochemical, and molecular assays using primary material

Fetal liver cells at E14.5 were used for colony-forming unit (CFU) assay and Western blot analysis.

Reconstituted and control mice were used for May–Grünwald–Giemsa staining, single-cell suspensions, flow cytometry, and histochemical analyses. For immune function assessments, ELISA was performed using cell supernatants or sera from immunized animals. Functional B cell assays included primary B cell stimulation, Ca<sup>2+</sup> measurements, RT-qPCR analysis, and RNA immunoprecipitation (RIP). Myeloid cell functional assays included generation of MDSCs (myeloid-derived suppressor cells), T-cell suppression assay, ROS production measurements, and Griess assay for nitric oxide (NO) production. Detailed protocols for all experiments are provided in the **Supplementary Methods**.

### Statistical analysis

Data were analyzed using Prism GraphPad 8.3.0 (GraphPad Software, USA). Statistical comparisons were performed using unpaired two-tailed t-tests. Results were considered statistically significant at p < 0.05.

## Data Availability

The original contributions presented in the study are included in the article/supplementary material. Further inquiries can be directed to the corresponding author.
