# Supplementary material for: YBX1 modulates humoral immunity through post-transcriptional regulation in B cells
Source: Front Immunol. 2025 Sep 10;16:1653073. doi: 10.3389/fimmu.2025.1653073 (PMC12457427; doi:10.3389/fimmu.2025.1653073)
Supplement: Supplementary file 1 [file DataSheet1.pdf]

Table S1

Organ	Cell subtype	Phenotype (single/live)	Absolute cell number per organ ± SD		% frequency ± SD	
			Ybx1 $^{+/+}$ (n=7-24)	*Ybx1* $^{-/-}$ (n=7-15)	*Ybx1* $^{+/+}$ (n=7-24)	*Ybx1* $^{-/-}$ (n=7-15)
**Bone Marrow 1x femur/1xtibea**	**Pro-B cells**	B220$^{+/low}$CD43^{+}	4.84x10e5 ± 2.21	5.17x10e5 ± 1.98	4.53 ± 0.99	4.27 ± 1.43
	**Pre-B cells**	B220^{low}CD43^{-}	9.84x10e5 ± 8.13	8.12x10e5 ± 5.57	10.62 ± 2.68	9.58 ± 4.22
	**Immature B cells**	B220^{+}IgD^{-}IgM^{+}	3.18x10e5 ± 3.15	1.53x10e5 ± 1.26	4.42 ± 1.86	3.96 ± 2.42
	**Recirculating B cells**	B220^{+}IgD^{+}IgM^{+}	8.08x10e5 ± 4.93	4.90x10e5 ± 3.21	6.40 ± 2.45	4.92 ± 1.67
	**Plasma B cells**	B220$^{+-/}$CD138^{+}	1.59x10e5 ± 1.51	2.54x10e5 ± 1.29	0.96 ± 0.52	1.05 ± 0.31
	**CD4^{+} T cells**	CD4^{+}	1.29x10e5 ± 0.35	3.15x10e5 ± 1.30	1.64 ± 0.45	2.44 ± 1.08
	**CD8^{+} T cells**	CD8^{+}	1.11x10e5 ± 0.45	2.55x10e5 ± 0.66	1.42 ± 0.58	1.97 ± 0.51
**Spleen**	**Naive mature B**	B220^{+}IgD^{+}IgM$^{+/low}$	8.42x10e6 ± 6.48	5.76x10e6 ± 3.76	35.64 ± 10.72	37.41 ± 4.97
	**Marginal zone B**	B220^{+}IgD$^{low/-}$IgM^{+}	6.18x10e5 ± 8.04	2.99x10e5 ± 2.97	8.13 ± 2.85	6.59 ± 1.77
	**Plasma B**	B220$^{+/-}$CD138^{+}	9.40x10e4 ± 5.86	5.60x10e4 ± 2.30	1.48 ± 0.89	1.44 ± 0.56
	**CD4^{+} T cells**	CD4^{+}	3.34x10e6 ± 6.10	3.10x10e6 ± 8.11	18.48 ± 3.36	21.03 ± 5.49
	**CD8^{+} T cells**	CD8^{+}	1.22x10e5 ± 1.03	1.36x10e5 ± 1.97	6.77 ± 1.57	9.05 ± 1.33

Organ	Myeloide cells in spleen	Phenotype (single/live/Lin^{-}CD11c^{-}CD11b^{+})	Absolute cell number per organ ± SD		% frequency ± SD	
			Ybx1 $^{+/+}$ (n=5)	*Ybx1* $^{-/-}$ (n=4)	*Ybx1* $^{+/+}$ (n=5)	*Ybx1* $^{-/-}$ (n=4)
**Spleen**	**Ly6C$^{-/low}$ Monocytes**	Ly6G^{-}SSC-H^{low}Ly6C^{low}	3.35x10e6 ± 0.85	2.82x10e6 ± 1.39	1.85 ± 0.47	1.91 ± 0.94
	**Ly6C$^{+/high}$ Monocytes**	Ly6G^{-}SSC-H^{low}Ly6C^{high}	2.79x10e6 ± 0.90	2.45x10e6 ± 0.87	1.54 ± 0.50	1.67 ± 0.59
	**Eosinophils**	Ly6G^{-}SSC-H^{high}	0.38x10e6 ± 0.06	0.82x10e6 ± 0.79	0.21 ± 0.03	0.55 ± 0.54
	**Neutrophils**	Ly6G^{+}	4.10x10e6 ± 1.27	3.57x10e6 ± 0.63	2.26 ± 0.70	2.42 ± 0.43

Organ	Myeloide cells in lung	Phenotype (single/live/CD45^{+})	% frequency ± SD	
			Ybx1 $^{+/+}$ (n=6)	*Ybx1* $^{-/-}$ (n=5)
**Lung**	**Inflammatory Monocytes (iMono)**	Ly6G^{-}IA/IE^{-}CD64^{+}CD11b^{+}CD11c^{-}Ly6C^{+}	1.08 ± 0.53	2.23 ± 0.96
	**Resident Monocytes (rMono)**	Ly6G^{-}IA/IE^{-}CD64^{+}CD11b^{+}CD11c^{+}Ly6C^{+}	0.94 ± 0.25	0.65 ± 0.28
	**Alveolar Macrophages (AM)**	Ly6G^{-}IA/IE$^{+/-}$CD64^{+}CD11c^{+}	11.70 ± 4.23	12.84 ± 2.52
	**Interstitial Macrophages (IM)**	Ly6G^{-}IA/IE$^{+/-}$CD64^{+}CD11c^{-}CD11b^{+}	0.25 ± 0.11	0.32 ± 0.07
	**Neutrophils**	Ly6G^{+}	11.03 ± 3.56	8.33 ± 3.39
	**Eosinophils**	Ly6G^{-}IA/IE^{-}CD64^{+}CD11b^{+}	4.14 ± 2.95	3.79 ± 4.24
	**CD11b^{+} Dendritic Cells (DC)**	Ly6G^{-}IA/IE^{+}CD64^{+}CD11b^{+}	0.24 ± 0.14	0.15 ± 0.08
	**CD11b^{-} Dendritic Cells (DC)**	Ly6G^{-}IA/IE^{+}CD64^{+}CD11b^{-}	0.21 ± 0.10	0.15 ± 0.11

Cell Subtype Quantification: Total cell numbers and frequencies of myeloid and lymphoid cell populations in the bone marrow, spleen, and lung of FLC-

Table S2

	Cell subtype	Phenotype (single/live)	Absolute cell number per organ ± SD			% frequency ± SD		
			Ybx1 ^{wild} (n=5)	*Ybx1* $^{+/+}$ (n=3)	*Ybx1* $^{-/-}$ (n=7)	*Ybx1* ^{wild} (n=5)	*Ybx1* $^{+/+}$ (n=3)	*Ybx1* $^{-/-}$ (n=7)
**Bone Marrow 1x femur/1x tibea**	**Pro-B cells**	B220$^{+/low}$CD43^{+}	5.81x10e5 ± 1.25	7.33x10e5 ± 2.61	5.22x10e5 ± 1.85	6.12 ± 1.71	5.02 ± 0.52	3.62 ± 0.36
	**Pre-B cells**	B220$^{+/low}$CD43-	8.55x10e5 ± 1.82	5.36x10e5 ± 2.85	4.65x10e5 ± 2.15	8.90 ± 1.90	3.56 ± 1.18	3.26 ± 1.02
	**Immature B cells**	B220^{+}IgD^{-}IgM^{+}	2.14x10e5 ± 1.01	1.59x10e5 ± 0.70	3.04x10e5 ± 0.84	13.68 ± 1.46	8.62 ± 0.83	10.60 ± 2.00
	**Recirculating B cells**	B220^{+}IgD^{+}IgM^{+}	7.30x10e5 ± 1.60	1.17x10e5 ± 3.92	5.51x10e5 ± 1.97	33.02 ± 4.23	48.97 ± 3.04	37.27 ± 3.85
**Spleen**	**Naive mature B**	B220^{+}IgD^{+}IgM$^{+/low}$	1.58x10e7 ± 0.73	3.27x10e7 ± 1.21	1.40x10e7 ± 0.49	79.92 ± 1.25	85.87 ± 1.33	78.11± 3.46
	**Marginal zone B**	B220^{+}IgD$^{low/-}$IgM^{+}	2.67x10e6 ± 1.17	2.62x10e6 ± 0.87	2.10x10e6 ± 1.06	13.74 ± 0.84	6.97 ± 0.76	15.89 ± 2.42
	**Plasma B**	B220$^{+/-}$CD138^{+}	1.21x10e5 ± 1.05	4.41x10e5 ± 3.36	0.85x10e5 ± 0.56	0.54± 0.28	1.08± 0.43	0.62 ± 0.15

Cell subtype quantification: Total cell numbers and frequencies of lymphoid cell populations in the bone marrow and spleen of FLC-reconstituted animals, measured six months post-transplantation. Data are presented as mean ± SD.

Table S3

B cell subtype	Phenotype	Absolute cell number in NP-KLH immunized mice ± SD		Absolute cell number in NP-FICOLL immunized mice ± SD	
		Ybx1 $^{+/+}$ (n=12)	***Ybx1*** $^{-/-}$ (n=6)	***Ybx1*** $^{+/+}$ (n=6)	***Ybx1*** $^{-/-}$ (n=5)
**Pro-B**	$B220^{+/low}CD43^{+}$	5.23x10e5 ± 1.98	4.37x10e5 ± 2.34	8.92x10e5 ± 2.42	7.13x10e5 ± 2.98
**Pre-B**	$B220^{low}CD43^{-}$	1.48x10e6 ± 5.51	8.08x10e5 ± 5.30	1.71x10e6 ± 5.89	1.85x10e6 ± 4.38
**Immature B**	$B220^{+}IgD^{-}IgM^{+}$	4.65x10e5 ± 1.76	2.55x10e5 ± 1.92	7.05x10e5 ± 1.65	6.49x10e5 ± 2.27
**Naïve mature B**	$B220^{+}IgD^{+}IgM^{+/low}$	7.56x10e6 ± 40.06	6.51x10e6 ± 42.09	1.19x10e7 ± 26.15	8.90x10e6 ± 41.14
**Marginal zone B**	$B220^{+}IgD^{low/-}IgM^{+}$	6.41x10e5 ± 3.72	8.47x10e5 ± 5.30	1.76x10e6 ± 7.07	1.31x10e6 ± 8.03
**Plasma**	$B220^{+/-}CD138^{+}$	1.32x10e5 ± 0.93	1.67x10e5 ± 1.68	1.06x10e5 ± 0.46	8.49x10e4 ± 0.23

B cell subtype quantification in immunized mice: Absolute numbers of B cell subtypes in the bone marrow and spleen of FLC-reconstituted mice immunized twice with either NP-KLH (adjuvanted with alum) or NP-FICOLL (in DPBS). Analysis was performed 7 weeks post-reconstitution and 2 weeks after the second immunization.

Table S4

Target	Fluorochrome	Klon	Isotype	Manufacturer
ß-ACTIN HRP	---	---	---	Cell Signaling Technology, Danvers, USA
B220	PE, Alexa Fluor® 647, Alexa Fluor® 488	RA3-6B2	Rat IgG2a, κ	BioLegend, San Diego, USA
BLNK	Alexa Fluor® 488	2B11	Mouse IgG2a, κ	BD Biosciences
CD117 (c-Kit)	Alexa Fluor® 647	2B8	Rat IgG2b, κ	BioLegend, San Diego, USA
CD11b	AmCyan	M1/70	Rat IgG2b, κ	BioLegend, San Diego, USA
CD11c	APC	N418	Armenian Hamster IgG	BioLegend, San Diego, USA
CD138	PE	7H11	Rat IgG1, κ	BioLegend, San Diego, USA
CD19	Alexa Fluor® 647, PE	6D5	Rat IgG2a, κ	BioLegend, San Diego, USA
CD20	APC-Cy7	SA275A11	Rat IgG2b, κ	BioLegend, San Diego, USA
CD22	PE	OX-97	Rat IgG1, κ	BioLegend, San Diego, USA
CD24	PerCP-Cy5.5	M1/69	Rat IgG2b, κ	BioLegend, San Diego, USA
CD25	PE-Cy7	PC61	Rat IgG1, λ	BioLegend, San Diego, USA
CD3ε	PE	145-2C11	Armenian Hamster IgG	BioLegend, San Diego, USA
CD4	PE	GK1.5	Rat IgG2b, κ	BioLegend, San Diego, USA
CD43	PE-Cy7	S11	Rat IgG2b	BioLegend, San Diego, USA
CD45	PerCP-Cy5.5	30-F11	Rat IgG2b, κ	BioLegend, San Diego, USA
CD45.1	PerCP-Cy5.5	A20	Mouse (A.SW) IgG2a, κ	BioLegend, San Diego, USA
CD45.2	PerCP-Cy5.5	104	Mouse (SJL) IgG2a, κ	BioLegend, San Diego, USA
CD64	PE-Cy7	X54-5/7.1	Mouse IgG1, k	BioLegend, San Diego, USA
CD8	APC	53-6.7	Rat IgG2a, κ	BioLegend, San Diego, USA
Donkey F(ab')2 Anti-Rat IgG H&L	Alexa Fluor® 488	---	---	Abcam, Cambridge, UK
F4/80	APC	BM8	Rat IgG2a, κ	BioLegend, San Diego, USA
FOXP3	Alexa Fluor® 647	MF-14	Rat IgG2b, κ	BioLegend, San Diego, USA
Goat@ rabbit IgG HRP	---	---	---	Cell Signaling Technology, Danvers, USA
IgD	PE-Cy7	11-26c.2a	Rat IgG2a, κ	BioLegend, San Diego, USA
IgM	PE, PerCP-Cy5.5	RMM-1	Rat IgG2a, κ	BioLegend, San Diego, USA
IKZF3	Alexa Fluor® 647	8B2	Mouse IgG1, κ	BioLegend, San Diego, USA
Ki-67	---	SolA15	Rat IgG2a, κ	eBioscience/Thermo Fisher Scientific
Lineage	Alexa Fluor® 488	145-2C11, RB6-8C5, M1/70, RA3-6B2, Ter-119	Armenian Hamster IgG, Rat IgG2b, Rat IgG2a	BioLegend, San Diego, USA
Ly6C	PE	1A8	Rat IgG2a, κ	BioLegend, San Diego, USA
Ly6G	APC-Cy7	HK1.4	Rat IgG2c, κ	BioLegend, San Diego, USA
Ms I-A/I-E	PE-Cy7	M5/114.15.2	Rat IgG2b, κ	BioLegend, San Diego, USA
NFATc1	Alexa Fluor® 488	7A6	Mouse IgG1, κ	BioLegend, San Diego, USA
NK-1.1	PE	PK136	Mouse IgG2a, κ	BioLegend, San Diego, USA
pPLCy2	Alexa Fluor® 647	PLCG2Y759-G3	Rabbit IgG, κ	Invitrogen
Sca-1	PE	D7	Rat IgG2a, κ	BioLegend, San Diego, USA
XBP-1S	Alexa Fluor® 647	Q3-695	Mouse IgG1, κ	BD Biosciences
YBX1	---	EP2708Y	Rabbit IgG	Abcam, Cambridge, UK

Antibodies used for B cell subtype identification by Flow cytometry and Western blot analysis: List of antibodies, including clone names, fluorochromes and manufacturer

Table S5

Target	Forward (5' - 3')	Reverse (5' - 3')
Actb	GGCTGTATTCCCCTCCATCG	CCAGTTGGTAACAATGCCATGT
Blnk	CAAGGTTCCCAGAAGCGGG	CATACCAGGGCTTACCGAGC
Cd22	ACAAAGCAGAGCCTGAGCTG	AGGTTGGAACGGTTTCTCCG
Cflar	TGCCTGAAGAACATCCACAGA	ATCTTGCTCCTTGGCTGGAC
Ebf1	AGCAATGGGATACGGACAGAA	CAGGGTTCTTGTCTTGGCCT
Igha	GGGAAGGATATAACCACCGTAAAC	AAGGAGGACAAGGAGGACAAGG
Ighg1, mature	CTTCCCAGCTGTCCTGCAGT	GGGGGGAAGATGAAGACAGATG
Ighg1, pre-mRNA	GGAAAAACCCATAGGCCTACACT	TTGTGTCTTCCTTGCTTTGGG
Ighm, membran	TCCTCCTGAGCCTCTTCTAC	CCAGACATTGCTTCAGATTG
Ighm, pre-mRNA	TGGACTCTGACCAGGGCTGT	GGAGACACAGCCAAGGCTAACTT
Ighm, secreted	CACACTGTACAATGTCTCCCT	AAAATGCAACATCTCACTCTG
Ikzf1	GGCTTTCGGGATCCCTTTGA	TCCACTCCCAACATTGTCCG
Ikzf3	CAACCGACTGTGGAGCTGAA	CATAGGGCACGTGTCTCTCC
Irf4	TCCCCATTGAGCCAAGCATAA	ACCAAAGCACAGAGTCACCT
Nfatc1	CCCGGAGTTCGACTTCGATT	AGGTGACACTAGGGGACACA
Nfatc3	TGGAGGCCACGAAATGATTGT	CCTGCACGGCAGATGTAACT
Nos2	CAGCTGGGCTGTACAAACCTT	CATTGGAAGTGAAGCGTTTCG
Plcg2	ATTCCTCCTGTCGCCAACTG	TGGTGCGTGTCGTAAAGGAA
Tcf3	CACCAGAACGCAACCTGAAC	CGGGCAGAGATATGGTGGTC
Tnf	CAGGCAGGTTCTGTCCCTTT	TCGCGGATCATGCTTTCTGT
Xbp1	ACACGCTTGGGAATGGACAC	CCATGGGAAGATGTTCTGGG
Ybx1	GTTGAAGGAGAAAAGGGTGCG	ATGGTTACGGTCTGCTGCAT

Primers used in RT-qPCR: List of forward and reverse primer sequences used for quantitative real-time PCR (RT-qPCR) analysis. All primers were synthesized by Merck, Darmstadt, Germany.
