# Supplementary material for: YBX1 modulates humoral immunity through post-transcriptional regulation in B cells
Source: Front Immunol. 2025 Sep 10;16:1653073. doi: 10.3389/fimmu.2025.1653073 (PMC12457427; doi:10.3389/fimmu.2025.1653073)
Supplement: Supplementary file 2 [file DataSheet2.pdf]

Figure S1

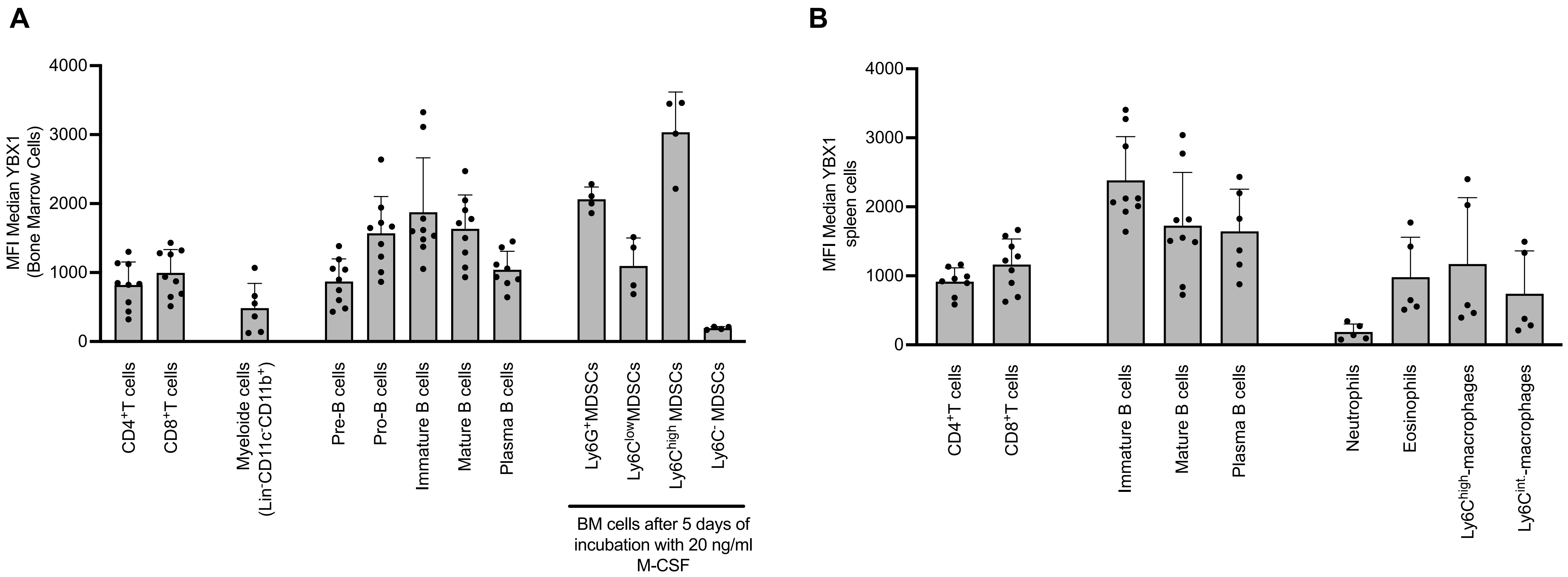

YBX1 expression in hematopoietic cells of the bone marrow (A) and spleen (B). Median fluorescence intensity (MFI) of YBX1 expression in live single-cell populations is shown. Data represent n = 4–9 mice per group.

Figure S2

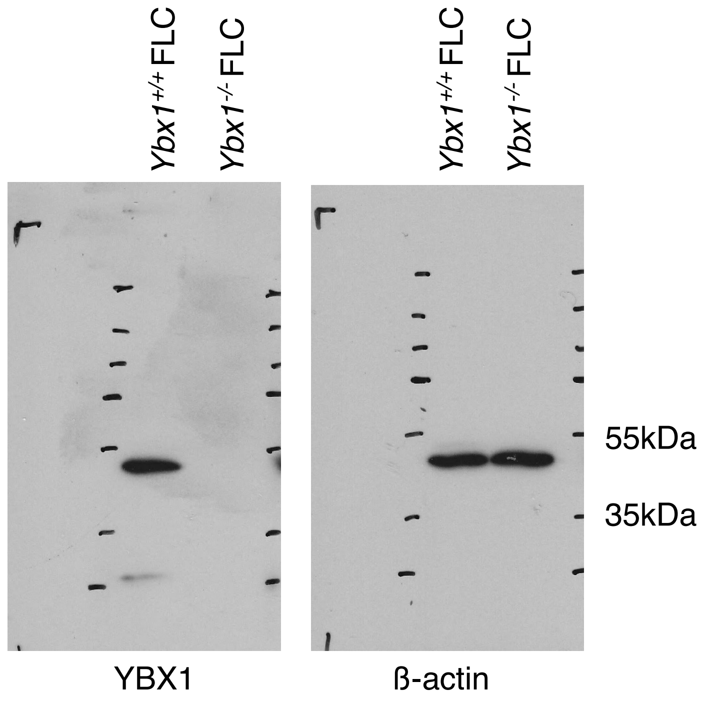

Western blot analysis of fetal liver cells from *Ybx1*$^{+/+}$ and *Ybx1*$^{-/-}$ embryos. Protein expression of YBX1 and the loading control β-actin is shown. Lysates were prepared from E14.5 fetal livers.

Figure S3

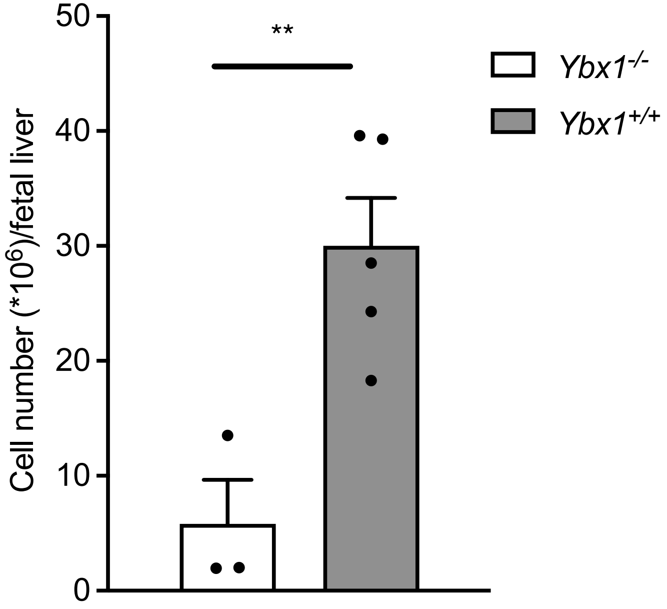

Total cell number per fetal liver in *Ybx1* $^{+/+}$ and *Ybx1* $^{-/-}$ embryos. Bars represent mean ± SD. Each data point corresponds to one individual fetal liver.

Figure S4

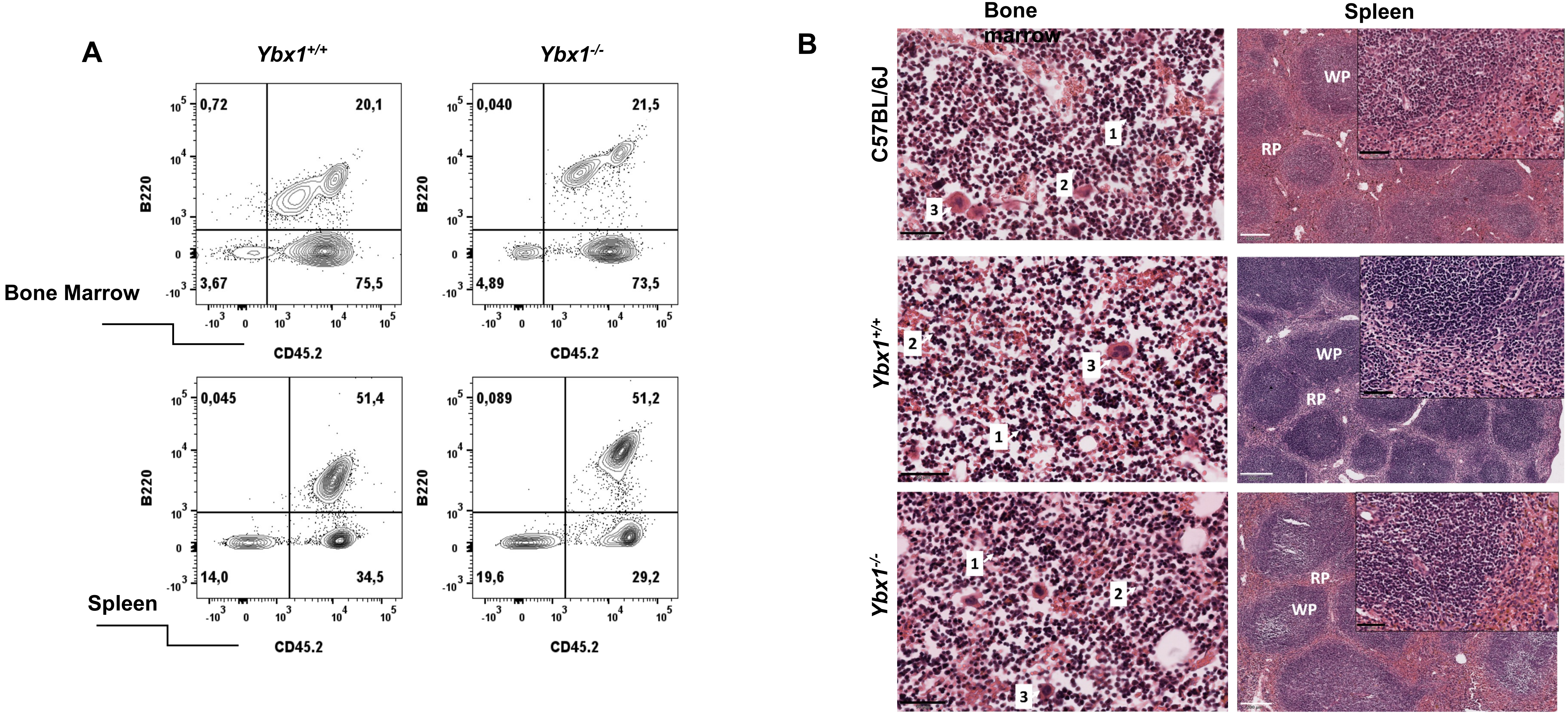

A. Donor-derived B Cells: Representative flow cytometry plots showing the frequencies of donor-derived (CD45.2$^+$) B220$^+$ B cells in the bone marrow and spleen eight weeks post-transplantation.

B. Histological analysis: Representative histological sections of bone marrow and spleen from untreated eight-week-old C57BL/6J mice and mice reconstituted with either *Ybx1*$^{+/+}$ or *Ybx1*$^{-/-}$ fetal liver cells, analyzed at eight weeks post-transplantation. White arrows indicate erythroid precursors (1), myeloid precursors (2), and megakaryocytes (3) in the bone marrow. Spleen sections show well-organized red pulp (RP) and white pulp (WP). Scale bars: black 50 µm; white 200 µm.

Figure S5

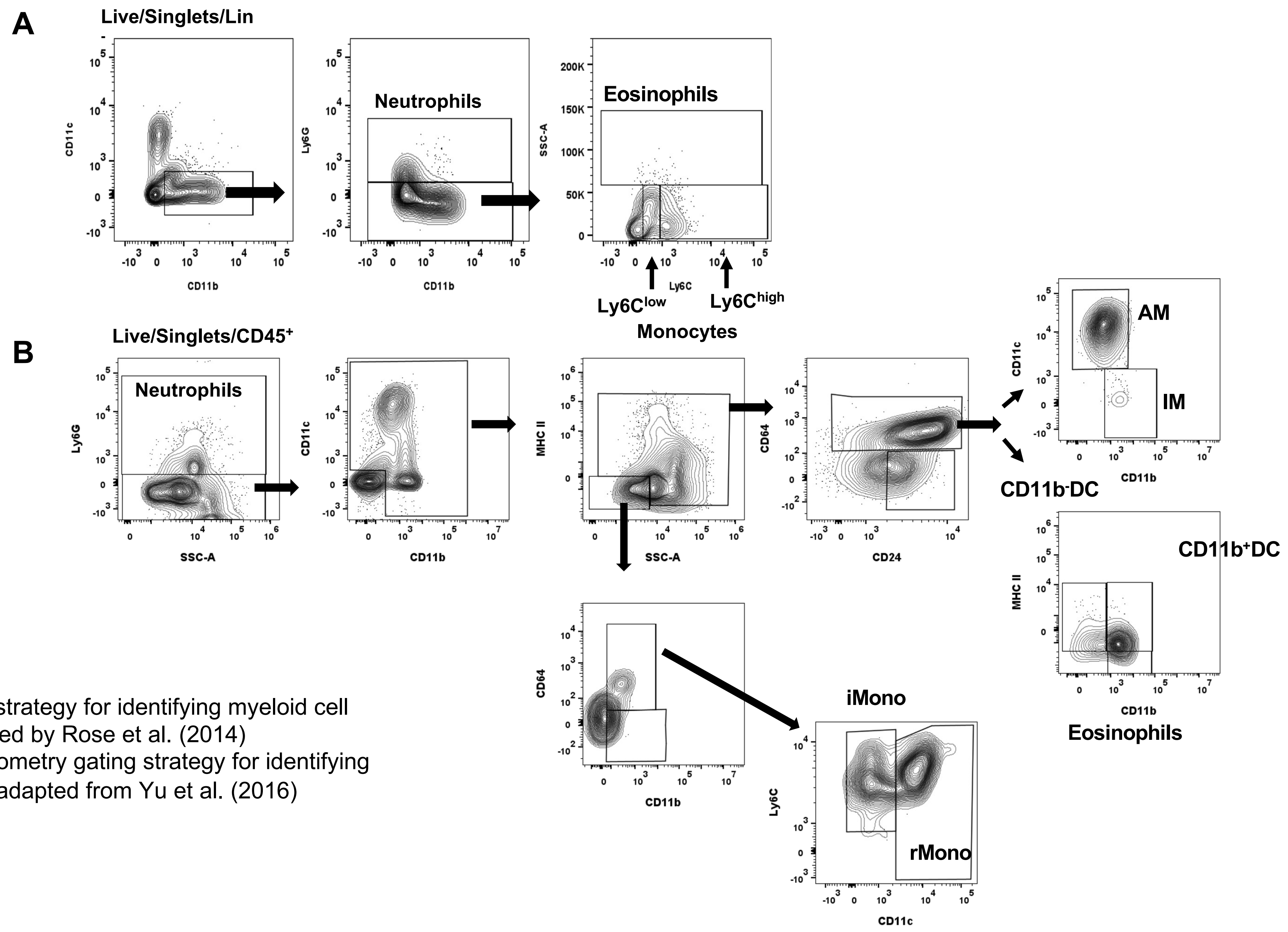

A. Myeloid Gating in Spleen: Gating strategy for identifying myeloid cell subtypes in the spleen, as described by Rose et al. (2014)
B. Myeloid Gating in Lungs: Flow cytometry gating strategy for identifying myeloid cell subtypes in the lung, adapted from Yu et al. (2016)

Figure S6

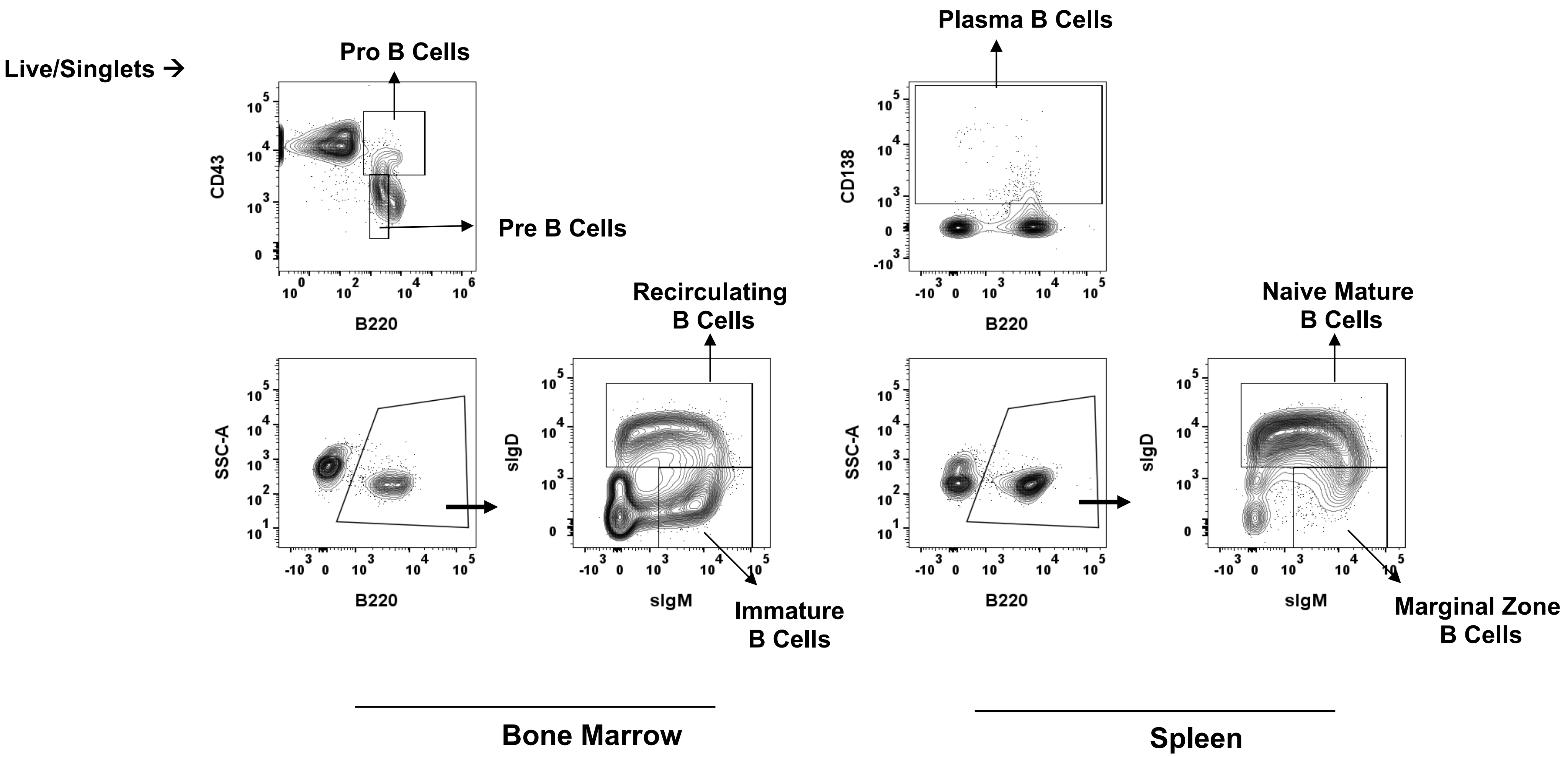

Gating strategy for the identification of B cell subtypes in bone marrow and spleen.

Figure S7

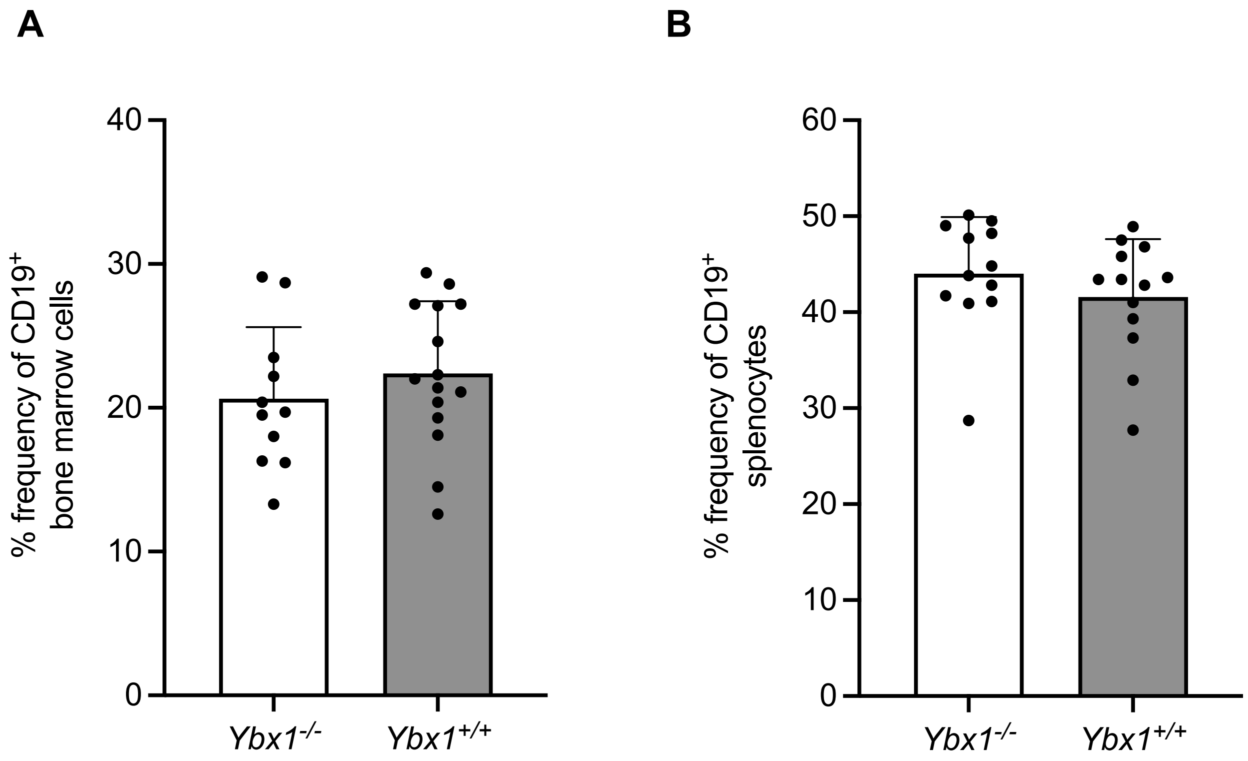

Frequencies of $CD19^{+}$ B cells in the bone marrow (A) and spleen (B) of *Ybx1*$^{+/+}$ and *Ybx1*$^{-/-}$ reconstituted mice 8 weeks after transplantation with fetal liver cells. Bars represent mean ± SD (n = 9–15 mice per group).

Figure S8

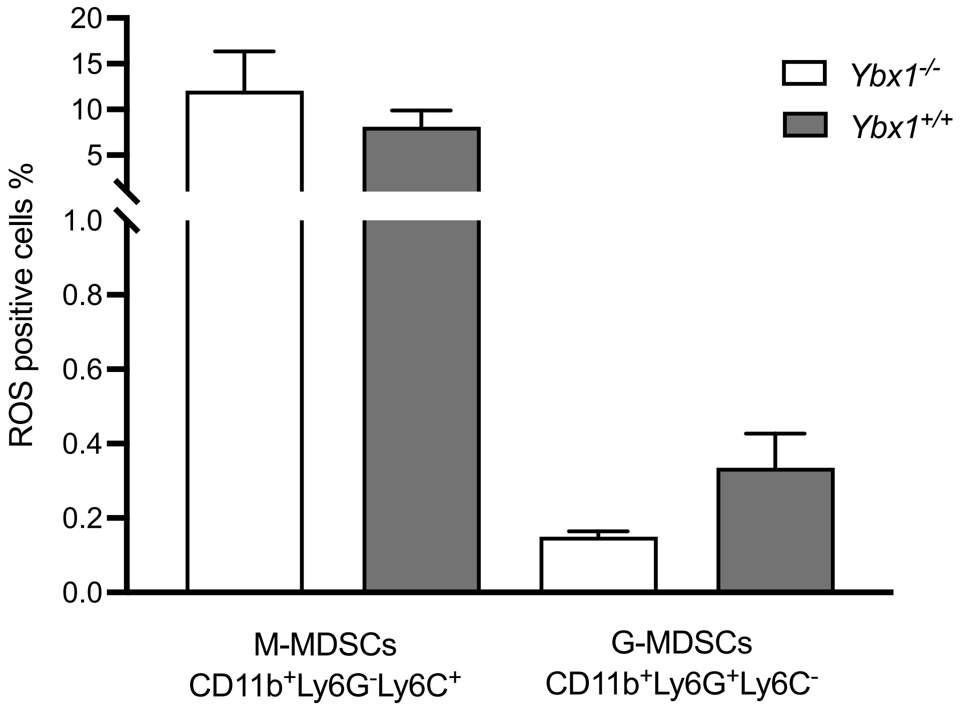

ROS production in MDSCs: Frequency of ROS-producing MDSCs after 24-hour stimulation with LPS and IFN-γ.

Figure S9

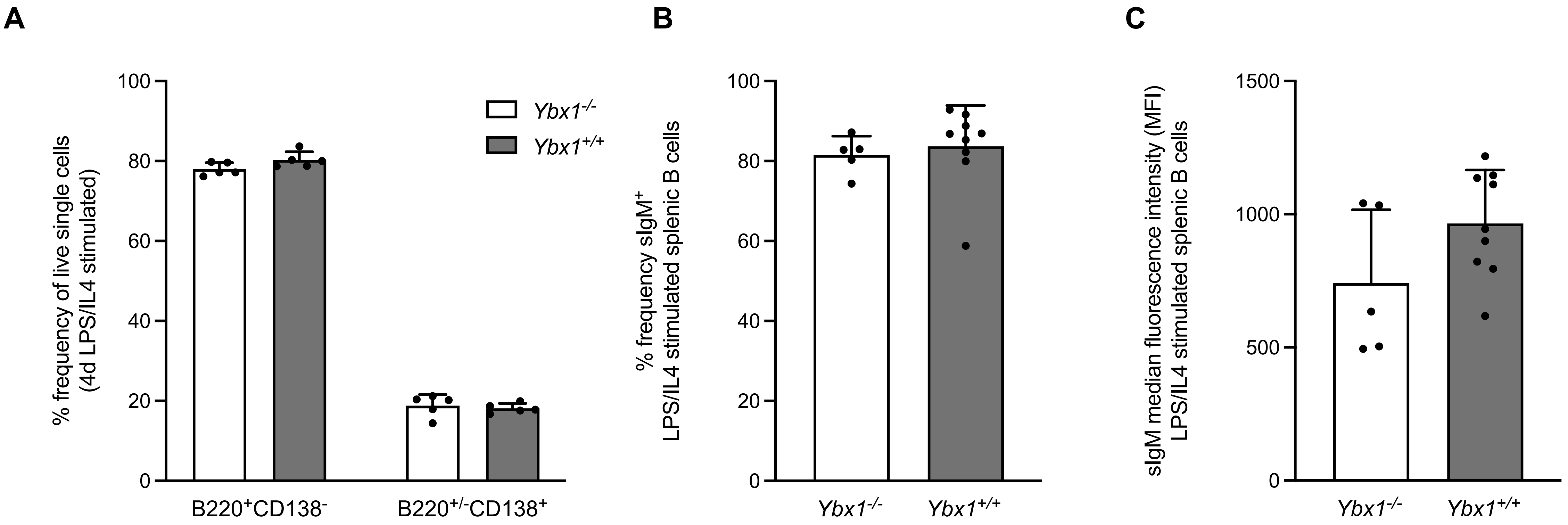

A. Frequency of live, single B220⁺ CD138⁻ and B220⁺/⁻ CD138⁺ splenocytes after 4 days of stimulation with LPS and IL-4 with a *Ybx1*⁺/⁺ or *Ybx1*⁻/⁻ genotype

B. Frequencies of IgM⁺ B cells after 4 days of cultivation with LPS and IL-4.

C. Flow cytometric analysis of surface IgM expression on Ybx1⁺/⁺ and Ybx1⁻/⁻ splenocytes, with median fluorescence intensity (MFI) shown as mean (n = 5–9). Each datapoint represents an individual mouse. Bars show mean values.

Figure S10

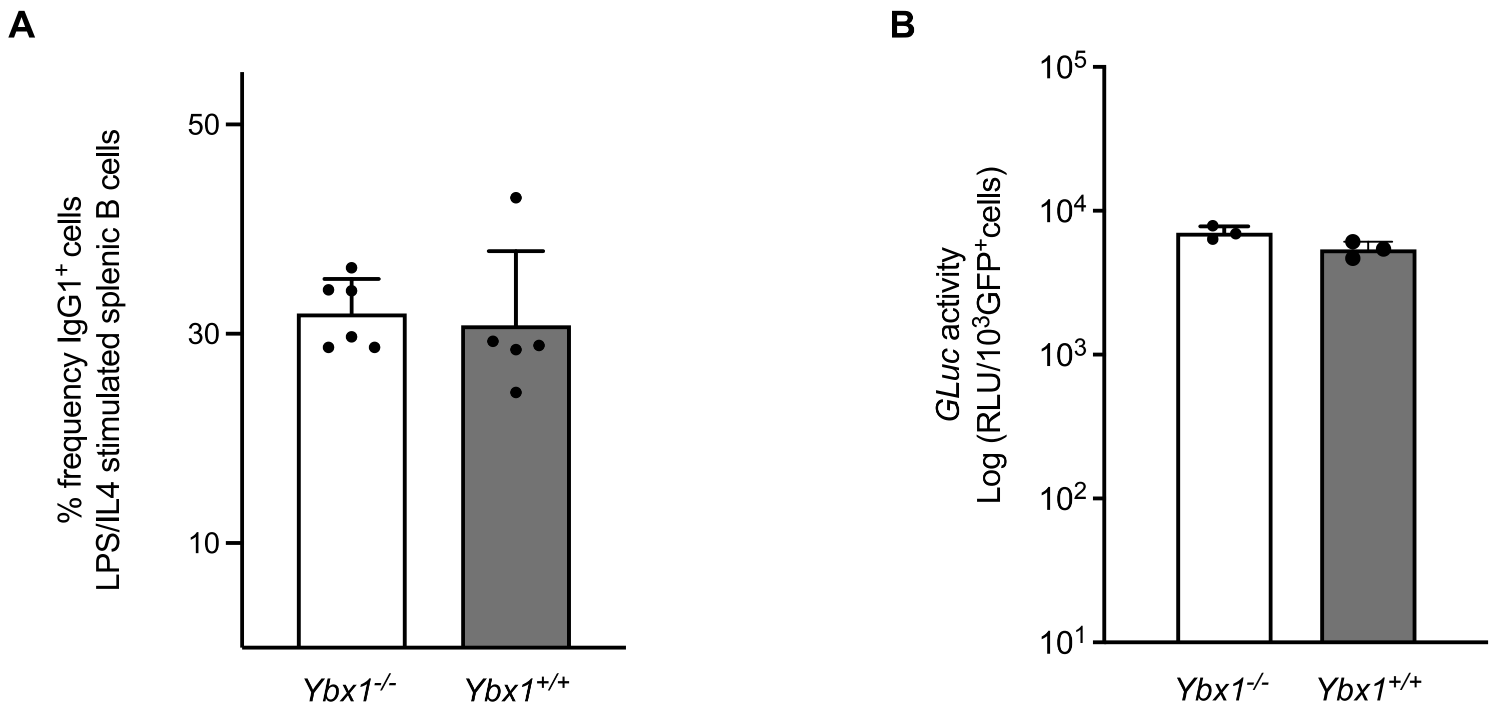

A. Cell Subtype Frequencies Post-Stimulation: Frequency of live, single B220^{+} B cells expressing IgG1 after 4 days of stimulation with LPS and IL-4, as determined by flow cytometry. Bars represent mean frequencies from individual mice.

B. GLuc Activity in Supernatant of Modified MEFs: Gaussia luciferase (GLuc) activity in the supernatant of modified mouse embryonic fibroblasts (MEFs) derived from Ybx1$^{-/-}$ or Ybx1$^{+/+}$ embryos. MEFs were transduced with a lentiviral construct containing two expression cassettes: GLuc and eGFP. Three days post-transduction, cells were plated, and GLuc activity was measured in culture supernatants. Data are presented as the logarithm of relative light units (RLU) secreted per 10^3 GFP^{+} cells. Columns represent mean ± SD (n = 3).

Figure S11

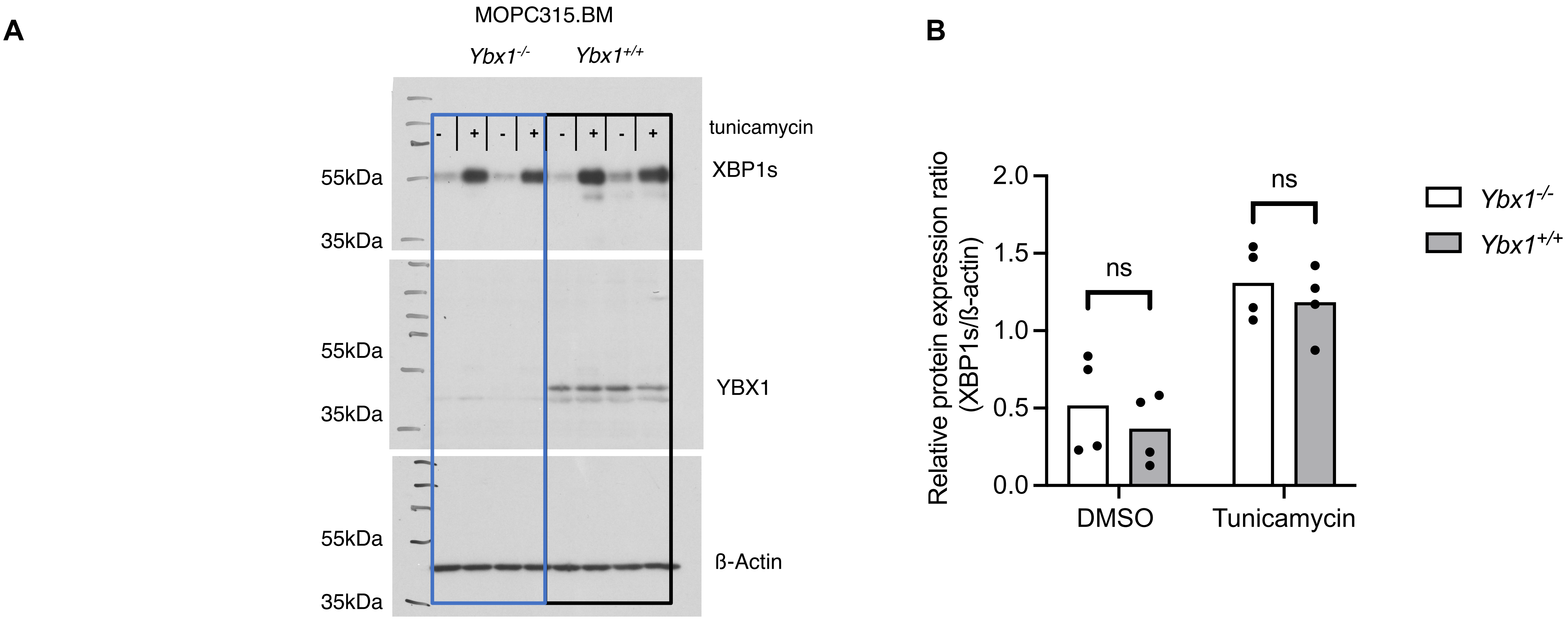

MOPC315.BM *Ybx1*$^{+/+}$ and *Ybx1*$^{-/-}$ cells were treated with 2 µg/mL tunicamycin or DMSO for 5 hours to induce ER stress. Protein lysates were analyzed by Western blot for YBX1 and spliced XBP1 (XBP1s). β-Actin served as a loading control. Quantification of XBP1s band intensity was done by ImageJ after scanning the film shown in A and two additional blots (Davarinejad, H. (2018). The blot in A shows two independent experiments for each genotype.

Figure S12

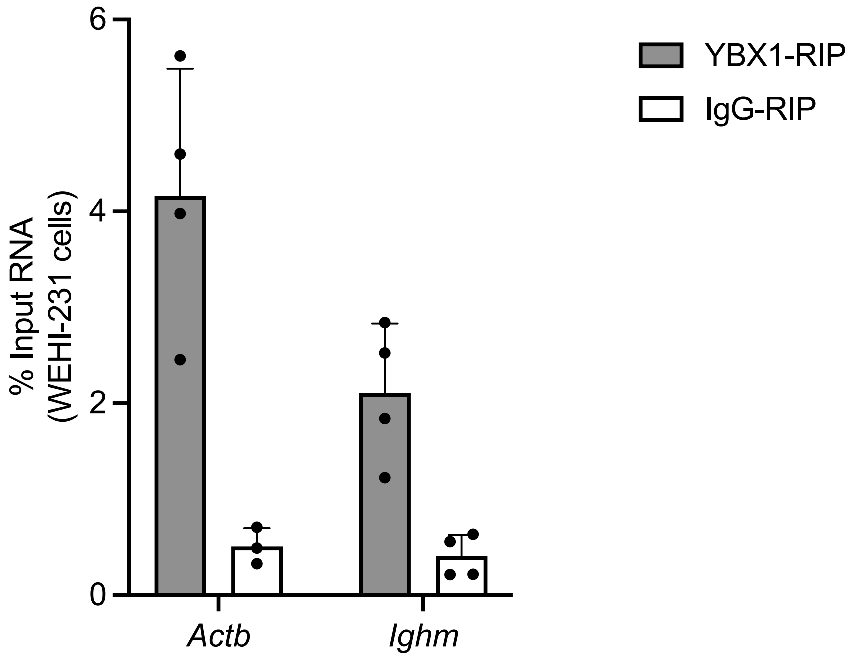

RNA immunoprecipitation (RIP) in WEHI-231 cells. Shown are the percentages of input RNA after RIP using anti-YBX1 or control IgG antibodies. qRT-PCR was performed to detect *Actb* (positive control) and *Ighm* transcripts. Data represent mean ± SD of technical replicates.

Figure S13

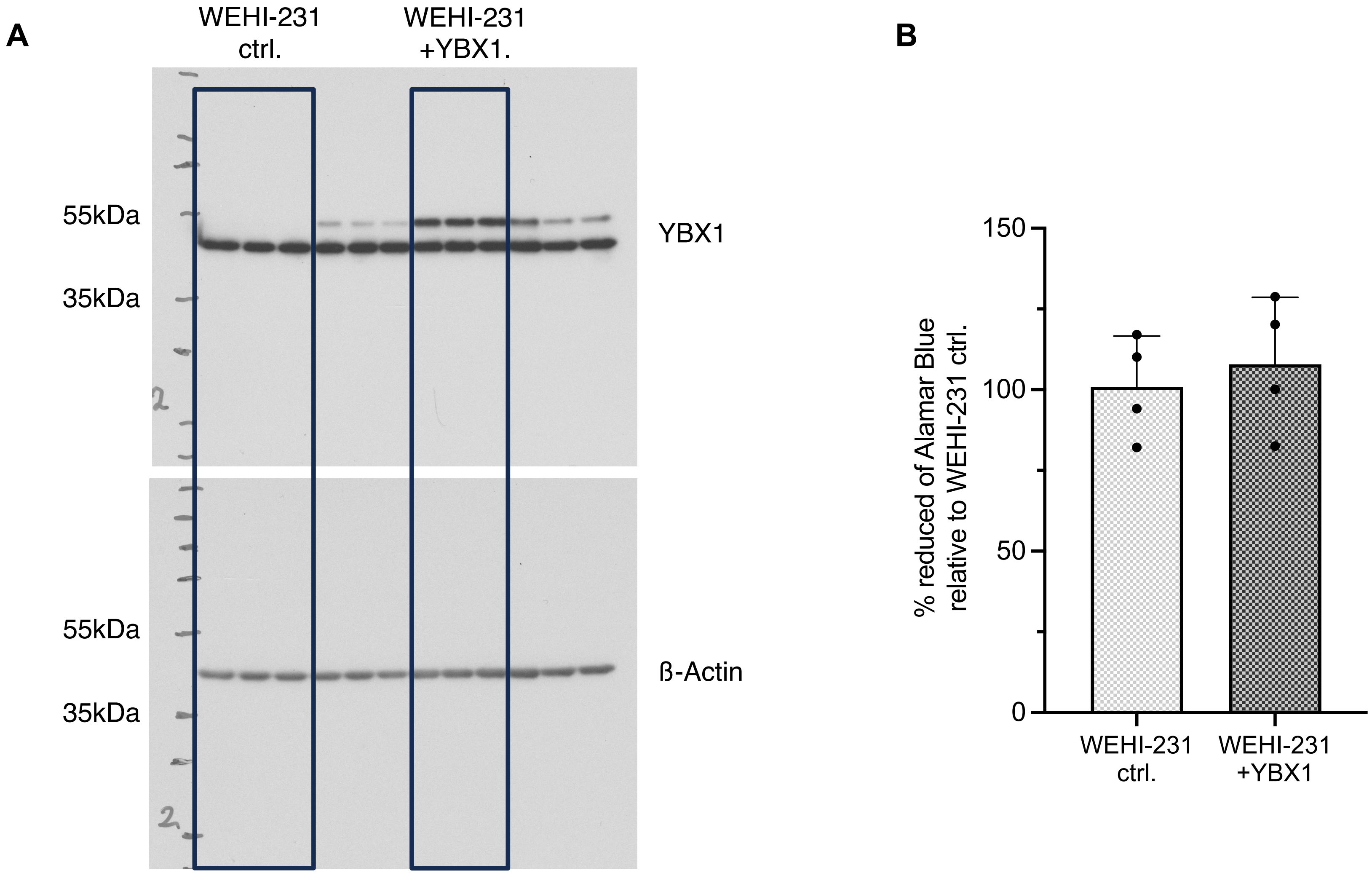

A. Western blot analysis of YBX1 expression in WEHI-231 cells. WEHI-231 cells were lentivirally transduced with a YBX1-overexpressing vector. The samples used in B are marked by boxes. β-Actin served as a loading control.

B. Relative metabolic activity as an indirect indicator of proliferation in WEHI-231 cells. Bar graph showing the percentage of Alamar Blue reduction in WEHI-231 ctrl. and WEHI-231 cells overexpressing YBX1. Data are normalized to the control cells and presented as mean ± SD.

Figure S14

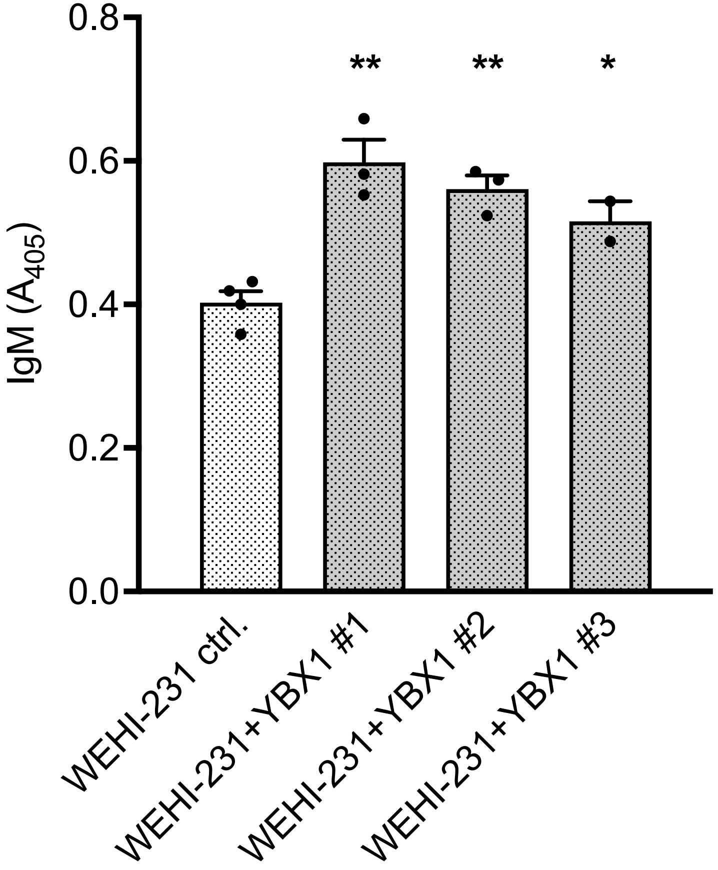

IgM detection in supernatants of WEHI-231 cells by ELISA. IgM levels in the supernatants of WEHI-231 ctrl. and three singe clones overexpressing YBX1 were analyzed. Detection was performed using an AP-conjugated secondary antibody, and absorbance was measured at 405 nm. Bars represent mean absorbance values ± SD.

Figure S15

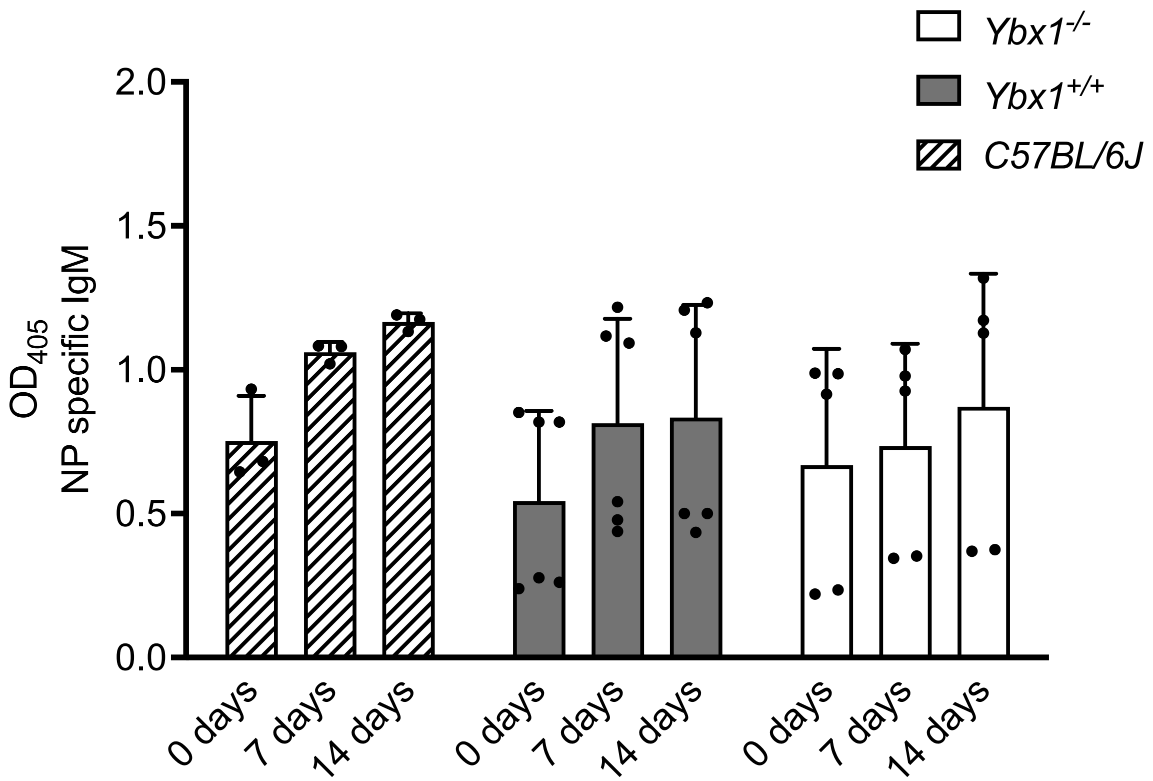

NP-specific IgM levels were measured by ELISA in the serum of reconstituted *Ybx1*$^{+/+}$ and *Ybx1*$^{-/-}$ mice at day 0, day 7, and day 14 after immunization with NP-Ficoll. Non-transplanted C57BL/6J mice served as immunocompetent controls. Antibody titers were assessed by absorbance at 405 nm (OD_{405}) using an AP-conjugated secondary antibody. Bars indicate mean ± SD.

Figure S16

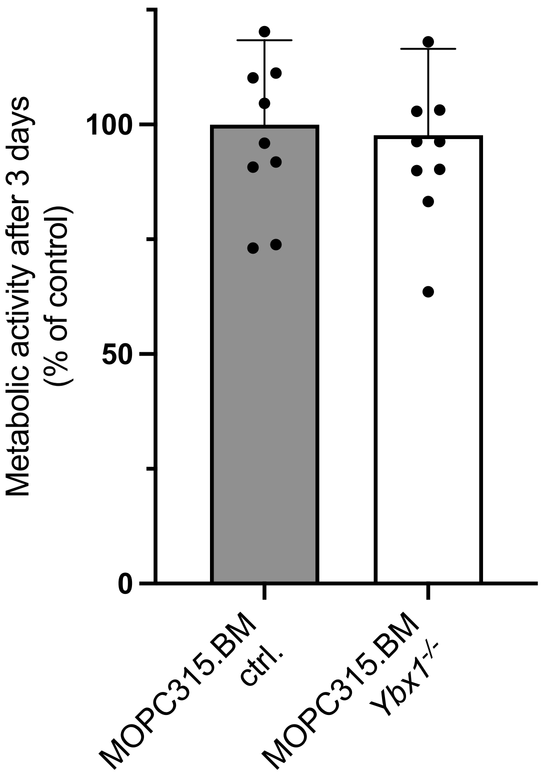

Metabolic activity in MOPC315.BM cells with or without YBX1 expression. Bar graph showing the percentage of MTT reduction in *Ybx1*$^{+/+}$ (ctrl.) and *Ybx1*$^{-/-}$ MOPC315.BM cells. Values are normalized to control (*Ybx1*$^{+/+}$) levels. MTT reduction reflects mitochondrial metabolic activity and is used as an indirect measure of cell viability and proliferation. Data are presented as mean ± SD.

Figure S17

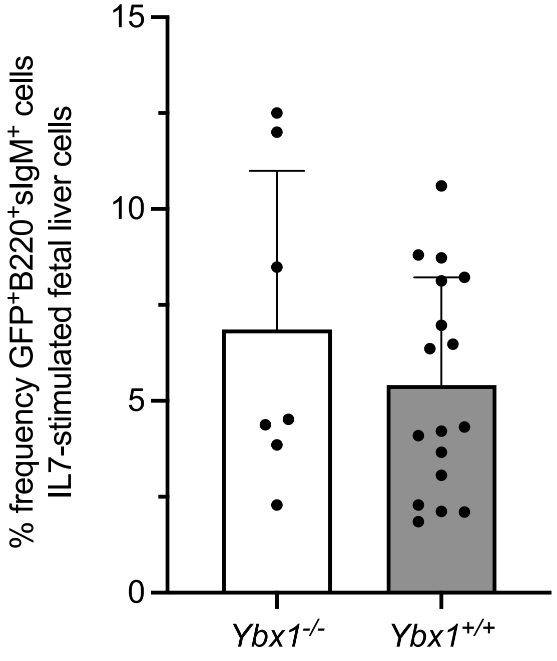

Frequency of GFP⁺B220⁺sIgM⁺ cells after IL-7 stimulation. Fetal liver cells from *Ybx1*$^{+/+}$ and *Ybx1*$^{-/-}$ embryos were lentivirally transduced with eGFP expressing vector and cultured in the presence of IL-7 . The percentage of GFP⁺B220⁺sIgM⁺ cells was determined by flow cytometry as a measure of early B cell differentiation. Bars indicate mean ± SD.

Figure S18

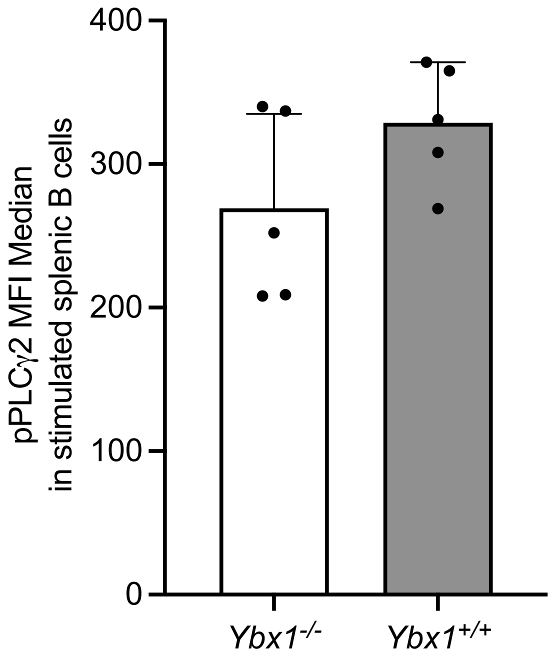

Median fluorescence intensity (MFI) of phospho-PLCγ2 in splenic B cells isolated from mice reconstituted with *Ybx1*$^{+/+}$ or *Ybx1*$^{-/-}$ fetal liver cells and stimulated *in vitro* with LPS and IL-4. Each data point represents an individual mouse.

Figure S19

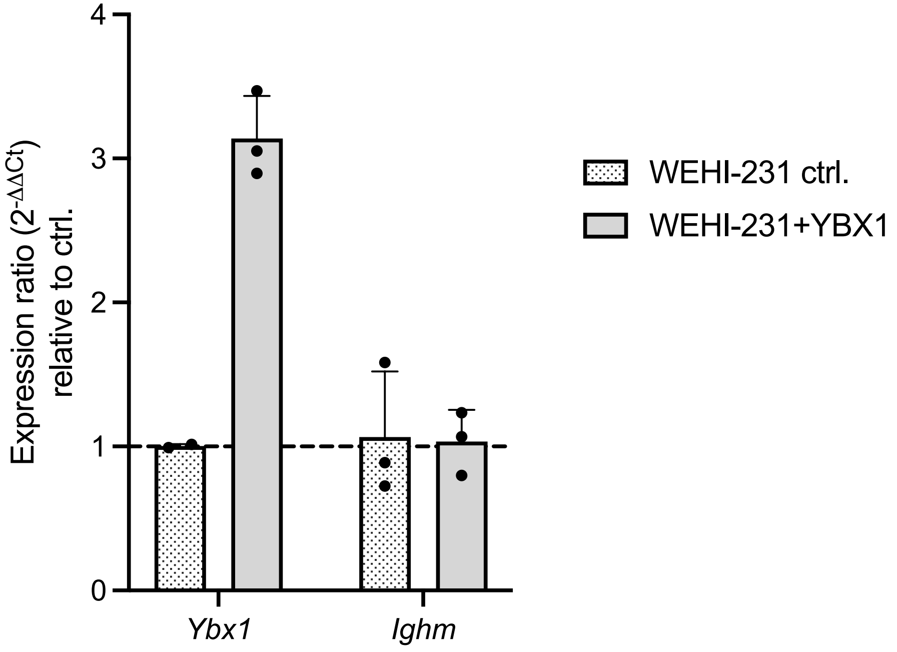

Relative mRNA expression of *Ybx1* and *Ighm* in WEHI-231 cells. Gene expression was analyzed by qRT-PCR and is shown as expression ratio ($2^{-\Delta\Delta Ct}$) relative to the control (WEHI-231 ctrl.). Bars represent mean ± SD of technical replicates.

Figure S20

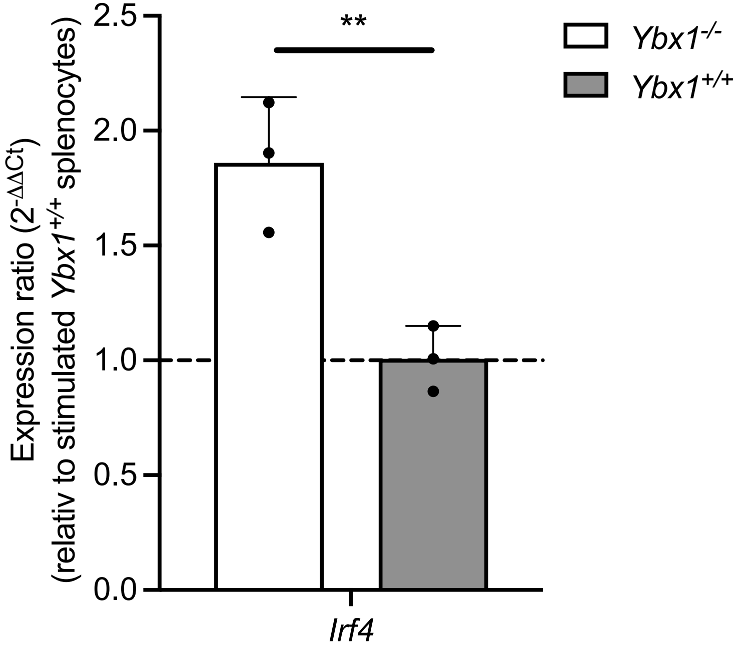

Quantitative RT-PCR analysis of *Irf4* mRNA in LPS/IL-4–stimulated splenic B cells from *Ybx1*$^{+/+}$ and *Ybx1*$^{-/-}$ mice. Expression levels are shown relative to *Ybx1*$^{+/+}$ controls. Bars represent mean ± SD. Each data point corresponds to an individual mouse.
