# Supplementary material for: YBX1 modulates humoral immunity through post-transcriptional regulation in B cells
Source: Front Immunol. 2025 Sep 10;16:1653073. doi: 10.3389/fimmu.2025.1653073 (PMC12457427; doi:10.3389/fimmu.2025.1653073)
Supplement: Supplementary file 3 [file DataSheet3.pdf]

Supplemental - Methods

Isolation of genomic DNA and PCR genotyping

Genomic DNA was isolated from embryonic or ear punch tissues using a NucleoSpin® Tissue kit (Macherey & Nagel, Germany). For genotyping, 100-250 ng gDNA was amplified by 35 cycles of PCR at 95 °C for 30 s, 60 °C for 30 s, and 72 °C for 1 min using the following primers:

Ybx1_F, `5'-agcgggtcacattcttacatag-3′`
Ybx1_R, `5'-aggaacggatacggtttcatca-3'` for *Ybx1* wild-type allele
Neo-3, `5'-caagaaggcgatagaaggcg-3'` and
Neo-5, `5'-attgcacgcaggttctccg-3'` for *Ybx1* knockout allele

Colony Forming Cell (CFC) Assay

FLCs ($2x10^4$ or $2x10^5$ cells for CFU pre-B analysis) were plated in a methylcellulose-based medium (R&D Systems, Germany) according to the manufacturer's instructions. Colonies were counted based on their morphology using a Nikon Eclipse TE200-U microscope (Nikon, Germany).

Western blot

Protein expression analysis was done as previously described (1). Antibodies are listed in Table S4.

May–Grünwald–Giemsa staining

Peripheral blood smears were air-dried for 1–2 hours and incubated in May–Grünwald solution (Roth, Germany) for 2 minutes. After two brief washes in tap water (1 minute each), slides were stained in Giemsa solution (Roth, 1:6 in 6.4 mM disodium hydrogen phosphate, 2.8 mM potassium dihydrogen phosphate) for 20 minutes. Slides were washed, air-dried, and analyzed under a Nikon Eclipse TE200-U microscope using a 60x oil immersion objective.

Single-cell suspensions

Spleens and lymph nodes were dissected, transferred into cell strainers, and mashed using a sterile syringe plunger. Bone marrow was flushed from femurs and tibias using PBS and a 5-mL syringe with a 24-gauge needle. Lung cells were isolated following the protocol of Yu et al. (2). Cell suspensions were filtered through a 70-µm cell strainer, and red blood cells were lysed using ACK buffer (154.4 mM ammonium chloride, 10 mM potassium bicarbonate, and 97.3 µM EDTA tetrasodium salt) before resuspension in cell-culture medium (RPMI, 10% FCS, 100 U/mL Pen-Strep, 0.1 µM β-Mercapto-ethanol).

Flow cytometry

Single-cell suspensions were stained using the Zombie Violet™ Kit (BioLegend, USA) for live/dead cell discrimination, followed by 5% rat serum blocking. Cells were stained with fluorochrome-conjugated antibodies (Table S4) and analyzed on a FACS Canto II or FACS Calibur cytometer (BD Biosciences, Germany). "Fluorescence minus one" (FMO) controls were used when necessary. Intracellular staining was performed using Fix/Perm buffer (3% formaldehyde, 0.1% saponin, 0.5% Tween-20) and Perm buffer (1% BSA, 0.5% saponin, 0.5% Tween-20) according to Albu et al (3). Data were analyzed using FlowJo software Version 10.10 (FlowJo, USA).

Myeloid cell functional assays

Monocyte differentiation: Bone marrow-derived monocytes were generated following Francke et al. (4) using bone marrow cells from reconstituted animals and 20 ng/mL M-CSF (BioLegend, USA) in the cell-culture medium for 5 days.

ROS production: 100,000 MDSCs were stimulated with 10 ng/mL LPS (Escherichia coli O111:B4, Merck, Germany) and 10 ng/mL IFN-γ (BioLegend, USA) for 24 hours. Cells were washed and preincubated with 1 µM H_2DCFDA (Selleck Chemicals, Germany) and 300 nM PMA (phorbol-12 myristate-13 acetate, Merck, Germany) for 30 minutes before surface protein staining and flow cytometric analysis.

Griess assay for NO production: Performed as described by Youn et al. (5).

T-cell suppression: The assay was performed as previously described (6). Briefly, 2,500, 10,000, or 200,000 LPS/IL-4-stimulated MDSCs were co-cultured with 100,000 pooled splenocytes/lymph node cells in the presence of 1 µg/ml anti-CD3 and 1 µg/ml anti-CD28 antibodies (both from BioLegend, USA) for four days. After incubation, cells were stained for viability assessment using the Zombie Violet™ Kit (BioLegend, USA) and subsequently blocked with 5% rat serum for 5 minutes. Surface staining was performed using anti-mouse CD4-PE and anti-mouse CD8-APC antibodies (both from BioLegend, USA). For proliferation analysis, cells were fixed and permeabilized, followed by staining with rat anti-Ki-67 and donkey anti-rat IgG Alexa Fluor 488 as the secondary antibody. Each staining step was performed for 20 minutes at room temperature. Flow cytometric analysis was conducted immediately after staining.

Stimulation of splenic B cells

Naive splenic B cells were purified 8–10 weeks post-transplantation by a negative selection (Pan B Cell Isolation Kit II, Miltenyi Biotec, Germany) and plated ($4x10^6$ cells/mL) in cell-culture medium supplemented with 50 µg/mL LPS and 1000 U/mL IL-4 (BioLegend, USA) for 4 days.

Calcium measurements

Stimulated splenic B cells were loaded with 2.5 µM Fluo-4 AM and 1.25 µM FuraRed AM (both from Biomol, Germany) in RPMI medium (1% FCS) in the presence of 0.04% Pluronic F127 (Thermo Fisher, Germany) for 45 minutes at 37 °C. Next, the cells were treated with the Zombie Violet™ Kit for 10 minutes at room temperature and subsequently stained with anti-B220-APC (BioLegend, USA) for 20 minutes at room temperature in the dark. The Fluo-4/FuraRed ratio was acquired using a BD FACSCanto II flow cyto-meter (BD Biosciences, Germany), recording a 2-minute baseline before stimulation. BCR-specific calcium mobilization was induced by adding 25 µg/ml anti-IgM $F(ab')_2$ (Jackson ImmunoResearch, USA), followed by continuous recording for an additional 10 minutes. The Fluo-4/FuraRed ratio over time was analyzed using the FlowJo Kinetics Tool (FlowJo, USA). Peak values and area under the curve (AUC) were quantified using Prism GraphPad 8.3.

ELISA

96-well ELISA plates were coated overnight with 1 µg/ml goat anti-mouse Ig or 10 µg/ml NP-BSA (LGC, Biosearch/Biocat, Germany) in coating buffer (0.1 M Na_2CO_3, 0.02% NaN_3, pH 9.6) at 4 °C. Plates were blocked, incubated with serum or culture supernatants, washed, and bound antibodies were detected

with alkaline phosphatase-conjugated anti-mouse IgM, IgG1, IgG2b, or IgG3 (Southern Biotech/Biozol, Germany). Absorbance at 405 nm was measured using a Tecan microplate reader (Tecan, Switzerland).

Histochemistry

Paraffin-embedded tissue was stained with H&E (Carl Roth, Germany) following the manufacturer's instructions.

RT-PCR

RNA was extracted using the NucleoSpin RNA isolation kit (Macherey & Nagel, Germany) according to the manufacturer's protocol. cDNA was synthesized using the High-Capacity cDNA Reverse Transcription Kit (Thermo Fisher, Germany). Real-time PCR was performed using qPCR EvaGreen Master Mix (Bio&Sell, Germany) and 250 nM primers (listed in Table S5). The cycling protocol was as follows: initial activation for 15 min (95 °C), 40 cycles of 15 sec at 95 °C, and 40 sec at 61 °C on a StepOnePlusTM Real-Time PCR System (Thermo Fisher, Germany). Relative mRNA expression was normalized to *Actb* and analyzed using the $2^{-\Delta\Delta Ct}$ method.

RNA-Immunoprecipitation (RIP)

YBX1-bound RNA was isolated and analyzed as described by Peritz et al. (7). Lysates from 1.5×10^7 MOPC315.BM cells were incubated with either 2 µg rabbit anti-YBX1 antibody or 2 µg rabbit IgG control (Jackson ImmunoResearch Laboratories, USA).

The relative enrichment of RNA in the IP fraction was calculated using the formula:

$$\% \text{ Input} = 2^{-\Delta Ct} \times 100$$

where $2^{-\Delta Ct}$ represents the relative RNA amount in the IP fraction compared to the input fraction.

Cloning of lentiviral vectors

pHAGE-GLuc-eGFP was cloned by digesting of pHAGE-CMV-DsRed-UBC-eGFP (a gift from Darrell Kotton, Addgene plasmid # 24526) and replacing DsRed with PCR amplified Gaussia luciferase. pCDH-MSCV-HA-YB1-copGFP was cloned by NheI-digestion of pCDH-MCS-copGFP-MSCV (System Biosciences, USA) and ligation of PCR amplified HA-YBX1 coding sequences. Linearized backbones and fragments were ligated using Gibson Assembly Master Mix (NEB, USA).

Lentiviral transduction

Lentiviruses production and cell transduction were performed as previously described. (8)

Gaussia luciferase assay

Gaussia luciferase was measured using the Dual-Glo Luciferase Assay system (Promega, Germany) following the manufacturer's instructions. The relative light units (RLU) were detected in a MicroLumat LB 96 P Microplate Luminometer (Berthold Technologies, Germany).

Supplemental Methods References

1. Bommert KS, Effenberger M, Leich E, Kuspert M, Murphy D, Langer C, et al. The feed-forward loop between YB-1 and MYC is essential for multiple myeloma cell survival. Leukemia. 2013;27(2):441-50.

2. Yu YR, O'Koren EG, Hotten DF, Kan MJ, Kopin D, Nelson ER, et al. A Protocol for the Comprehensive Flow Cytometric Analysis of Immune Cells in Normal and Inflamed Murine Non-Lymphoid Tissues. PLoS One. 2016;11(3):e0150606.

3. Albu DI, Califano D, Avram D. Flow cytometry analysis of transcription factors in T lymphocytes. Methods Mol Biol. 2010;647:377-90.

4. Francke A, Herold J, Weinert S, Strasser RH, Braun-Dullaeus RC. Generation of mature murine monocytes from heterogeneous bone marrow and description of their properties. J Histochem Cytochem. 2011;59(9):813-25.

5. Youn JI, Nagaraj S, Collazo M, Gabrilovich DI. Subsets of myeloid-derived suppressor cells in tumor-bearing mice. J Immunol. 2008;181(8):5791-802.

6. Eckert I, Ribechini E, Lutz MB. In Vitro Generation of Murine Myeloid-Derived Suppressor Cells, Analysis of Markers, Developmental Commitment, and Function. Methods Mol Biol. 2021;2236:99-114.

7. Peritz T, Zeng F, Kannanayakal TJ, Kilk K, Eiriksdottir E, Langel U, et al. Immunoprecipitation of mRNA-protein complexes. Nat Protoc. 2006;1(2):577-80.

8. Effenberger M, Bommert KS, Kunz V, Kruk J, Leich E, Rudelius M, et al. Glutaminase inhibition in multiple myeloma induces apoptosis via MYC degradation. Oncotarget. 2017;8(49):85858-67.

9. Davarinejad, H. (2018). Quantifications of Western Blots With ImageJ. Avaliable at: www.yorku.ca/yisheng/Internal/Protocols/ImageJ.pdf (accessed 18.8.25).
